# In Situ Characterization of Strontium Titanium Ferrite Perovskites for Application as Electrodes of Solid Oxide Cells

**DOI:** 10.1021/acsaem.5c03226

**Published:** 2026-01-23

**Authors:** Maria Carmenza Diaz Lacharme, Martina Marasi, Virginia Pérez Dieste, Belen Ballesteros, Alessandro Donazzi

**Affiliations:** † Department of Energy, 18981 Politecnico di Milano, Via Lambruschini 4, Milan 20156, Italy; ‡ ALBA Synchrotron Light Source, Carrer de la Llum 2-26, Cerdanyola Del Vallès, Barcelona 08290, Spain; § Catalan Institute of Nanoscience and Nanotechnology (ICN2), CSIC and the Barcelona Institute of Science and Technology, Campus UAB, Bellaterra, Barcelona 08193, Spain

**Keywords:** exsolution, In situ characterization, NAP-XPS, XRD, solid oxide cell, electrolysis, CO_2_

## Abstract

This study investigates the redox behavior, the surface composition, and the electrochemical performance of the Ni-doped Sr_0.95_(Ti_0.3_Fe_0.63_Ni_0.07_)­O_3_ (STF-Ni) and SrTi_0.3_Fe_0.7_O_3_ (STF) perovskites under conditions relevant to solid oxide cell applications. The effect of Ni doping on exsolution and on its reversibility is examined with synergistic characterization techniques. In situ XRD experiments (in 5% H_2_, up to 750 °C) reveal that FeNi and FeNi_3_ coexist in alloyed Ni–Fe nanoparticles and that the exsolved Ni is only partially reincorporated in the lattice of STF-Ni on reoxidation in air. In contrast, the reduction of STF leads to the segregation of nonalloyed metallic Fe particles. In situ near-ambient pressure XPS experiments (20 mbar, 550 °C) show that Sr segregates on both perovskites as SrO_*x*_ during reduction in pure H_2_, and that the exsolution of Ni and Fe enhances the segregation. Subsequent exposure to CO_2_ causes the formation of SrCO_3_ and compositional changes of the STF-Ni nanoparticles, which become richer in Ni due to the back-diffusion of Fe in the lattice. When applied as fuel electrodes of electrolyte-supported solid oxide cells, STF-Ni and STF exhibit distinct behaviors in H_2_ electro-oxidation and CO_2_ electrolysis. STF-Ni shows better performance than STF with 3% humidified H_2_ supply (450 vs 350 mW/cm^2^ at 0.5 V), while both electrodes achieve similar current density (−450 mA/cm^2^ at 1.4 V) in reversible CO_2_ electrolysis with a 50/50 CO/CO_2_ mixture. These performance differences primarily arise from the interaction with CO_2_, which causes SrCO_3_ formation, electrode passivation, and compositional modifications of the nanoparticles.

## Graphical abstract

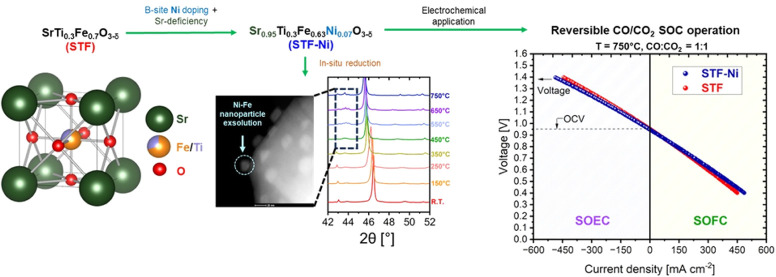

## 1 Introduction

Exsolution is a method to functionalize the surface of perovskite oxides via the formation of metal nanoparticles. In this process, a perovskite with reducible B-site transition or noble metal cations is exposed to reducing conditions, such that the B-site cations emerge to the surface in the form of nanoparticles.^1–3^ This method is gaining increasing attention, especially for the preparation of fuel electrodes for solid oxide cells (SOCs) and of catalysts for high-temperature reactions,^4–9^ thanks to the remarkable resistance to particles’ sintering. Compared to conventional deposition methods (e.g., CVD, infiltration, wet impregnation), exsolved nanoparticles show better resistance to agglomeration, coarsening, and clustering due to their partial embedding (socketing) into the perovskite lattice. A certain portion of the nanoparticle volume (up to 30% with Ni^10^) always remains submerged in the oxide lattice, such that anchorage is improved. The homogeneous spatial distribution of the exsolved nanoparticles also enhances the electrode’s tolerance to redox cycling and allows to remarkably cut the amount of active metal phase, similarly to what happens in electroless deposition.^11^ When processing carbon-based fuels, the socketed nanoparticle configuration suppresses the harmful tip-growth mechanism in favor of base-growth, enhancing the perovskite’s coke resistance.^2,3,10^ Ultimately, exsolution is the selective reduction of a metal oxide, which emerges up to the perovskite surface via phase separation due to a miscibility gap in the solid solution of the perovskite’s lattice, induced by exposure to appropriate conditions.^12^ Several factors simultaneously establish the nature of the exsolved nanoparticles and influence their size, population density, and composition. External parameters include the gas phase composition, temperature, applied electric potential, and duration of the exposure to the reducing conditions. Additionally, intrinsic parameters associated with the microscopic morphological features of the perovskite’s surface, such as smoothness, termination facets, cleaved planes, and local strain, affect the extent of the exsolution process and the composition of the nanoparticles.^3,10^ A quest remains to control their growth to tailor the catalytic and electrocatalytic activity of the resulting material.

Fe-substituted strontium titanates are prototypical platforms for the exsolution of Fe-alloyed nanoparticles. When doped, especially with Ni or Ru, these simple perovskites are commonly explored as electrodes of SOCs for steam electrolysis (SOECs) and fuel electro-oxidation (SOFCs). Since detrimental secondary phases evolve from stoichiometric formulations (A/B = 1) upon exsolution, nonstoichiometric formulations with A-site deficiency are utilized: in this case, the parent perovskite approaches the stoichiometric structure after losing the reducible cation, since the A-site deficiency helps in compensating for the formation of B-site vacancies.^13,14^ Accordingly, Sr_1–*x*_(Ti_0.3_Fe_0.7–*y*_Ni_*y*_)­O_3−δ_ and Sr_1–*x*_(Ti_0.3_Fe_0.7–*y*_Ru_*y*_)­O_3−δ_ (*x* ≤ 0.05 and *y* ≤ 0.07) are preferential.^12,14–20^ Open questions, however, concern the stability of the parent phase obtained after exsolution: depending on the reduction conditions and the initial stoichiometry, some authors^12,21^ observe the transition to adverse Ruddlesden–Popper (RP) phases, which present lower electrocatalytic activity and induce mechanical instability due to lattice expansion. Stabilization can be achieved by limiting the reduction temperature and by increasing the equivalent pO_2_: accordingly, Sr_0.95_Ti_0.3_Fe_0.7_O_3−δ_ is reported to remain stable at 850 °C for pO_2_ larger than 6.5 × 10^–18^ bar (i.e., with pH_2_/pH_2_O equal to 1), whereas the decomposition to RP becomes much faster for H_2_ richer conditions, longer exposure time, and different Fe/Ti ratios.^21^ The amount of dopant that exsolves and reaches the surface also depends on multiple factors. On the one hand, not all the dopant that has reduced to the metal state emerges up to the surface; rather, it can remain embedded in the lattice as a consequence of the interplay between nucleation, lattice strain, and mass transport. On the other hand, not only the dopant metal exsolves, but also the main B-site element does, leading to alloys or core–shell structures:^22^ this suggests that the parent perovskite possibly becomes richer or leaner in one of its constitutive elements, questioning the beneficial mechanism entailed by A-site deficiency. While Ni and Ru exsolve almost completely,^21^ alloys with Fe are also formed (with approximately 50/50 Fe/Ni content in strontium iron titanates^14,20^ ). Hence, considering the complexity of the exsolution process, its reversibility via redox cycling is also debated. Although complete redissolution of the nanoparticles (typically 30–50 nm in size) and back-diffusion are unlikely, stoichiometric perovskites are sometimes reported to restore their original structure upon reoxidation with full readsorption of the nanoparticles, while substoichiometric perovskites generally do not.^17,23,24^

The effect of the applied electric potential also modifies the extent of exsolution and the stability of the perovskite. Several effects can be triggered, which span from the increase of the population density of the nanoparticles to the modification of their composition.^25,26^ Several authors achieve significant electrochemical performance in H_2_ electro-oxidation and steam electrolysis. A variation of the maximum power density from 1.3 to 0.3 mW/cm^2^ passing from 850 to 750 °C is reported by the group of Barnett^20^ for SOFCs mounting Sr_0.95_(Ti_0.3_Fe_0.63_Ni_0.07_)­O_3−δ_ (STF-Ni) as the fuel electrode, when operated with 3% humidified H_2_. The same group^18^ shows that STF-Ni-based SOECs reach 1.5 A/cm^2^ at 800 °C in steam electrolysis and achieve stable operations at 750 °C for 300 h under alternating polarization between −0.5 and +0.5 A/cm^2^. Similarly, Han et al.^15^ find that SOECs with an STF-Ni fuel electrode yield 0.95 A/cm^2^ in steam electrolysis at 800 °C and 1.3 V, when supplied with a 50% H_2_ 50% H_2_O mixture. Notably, this performance is almost insensitive to the nature of the nanoparticles (compared to Cu–Fe, Ru–Fe, and Co–Fe), rather it is only dependent on the presence of alloyed Fe. Scarce activity and deactivation are instead observed during CO_2_ electrolysis, where STF-Ru seems preferential. Our group has also investigated the electrocatalytic performance of Sr_0.95_(Ti_0.3_Fe_0.63_Ni_0.07_)­O_3−δ_ fuel electrode in electrolyte-supported SOCs.^27^ STF-Ni has shown stable performance in the steam electrolysis of a 50/50 H_2_/H_2_O mixture at 750 °C with no degradation during 100 h operations under alternate cycles between 1.2 and 0.85 V. Irreversible and quick deactivation was instead observed in CO_2_ electrolysis with a 50/50 CO/CO_2_ mixture. In this work, by the application of in situ X-ray diffraction (XRD) and in situ near atmospheric X-ray photoelectron spectroscopy (NAP-XPS) performed at the Alba synchrotron in Barcelona (Spain), we investigate the exsolution and reoxidation behavior of STF-Ni and also of the undoped SrTi_0.3_Fe_0.7_O_3‑δ_ (STF) perovskite, which is taken as a close approximation of the parent phase obtained after exsolution of STF-Ni. The application of synchrotron techniques enables us to detect the presence of nascent secondary phases such as simple oxides that potentially form even from the initial stages of exsolution or survive after reoxidation. The consequences of exposing both materials to CO_2_ are also explored. By application of Near Edge X-ray Absorption Fine Structure (NEXAFS), TEM, and thermogravimetric analyses, we characterize the reduction properties and the uptake of CO_2_ and correlate them with the electrochemical performance as SOCs.

## 2 Materials and Methods

### 2.1 Powder Synthesis

The solid-state synthesis (SSS) method was used to prepare the perovskite powders. The precursors used for the synthesis of SrTi_0.3_Fe_0.7_O_3_ (STF) were SrCO_3_ (Carlo Erba Reagents), TiO_2_ (Tronox CrystalACTiV), and Fe_2_O_3_ (Sigma-Aldrich). Ni­(NO_3_)_2_·6 H_2_O (Sigma-Aldrich) was added to the base STF precursors to synthesize Sr_0.95_(Ti_0.3_Fe_0.63_Ni_0.07_)­O_3_ (STF-Ni) powders according to the required stoichiometry. The precursors were ball-milled for 96 h using zirconia spheres as the grinding medium, with 180 g of 0.5 cm spheres and 60 g of 1 cm spheres per 20 g of precursor. Based on the average sphere weight, this corresponds to approximately 430 small spheres and 20 large spheres. Two different sphere sizes were used to combine the effect of the larger spheres, which promoted the breakdown of coarse agglomerates, with the finer milling action of the smaller spheres, which ensured homogeneous particle refinement. Milling was performed as a wet process in 50 mL of ethanol, inside sealed Nalgene bottles, at a rotation speed of 95 rpm. Once the milling step was completed, the powder and solvent mixture was dried at room temperature for 72 h and then calcined at 1200 °C for 10 h at 1 K/min heating and cooling rate. The calcined powders were then ball-milled for an extra 96 h to achieve a finer particle size.

### 2.2 Structural and Chemical Characterization

In situ XRD experiments were performed using a synchrotron-based high-resolution powder diffraction system at the MSPD beamline of the ALBA synchrotron equipped with a MYTHEN-II detector. The source used synchrotron radiation with a wavelength of 0.4139 Å. The diffraction patterns were collected in the 0.55–60° 2θ range with a step size of 0.006°. Conversion of the patterns to the standard Cu wavelength (1.5406 Å) was done using Bragg’s law. The samples were prepared by packing powders into quartz capillaries (Hilgenberg). The capillaries had an outer diameter of 1 mm and an inner diameter of 0.58 mm. Heating was achieved using an external hot air blower, with temperature controlled up to 750 °C at a ramp rate of 5 K/min. Two types of atmospheres were used for the experiments: a safety mixture with 5% H_2_ in N_2_ that simulated reduction and testing of a SOC under H_2_, and a 10% O_2_ in N_2_ mixture. These gas compositions were selected to investigate the exsolution process in STF-Ni and its reversibility under oxidizing conditions, enabling a direct comparison with the baseline behavior of the undoped parent perovskite STF. Both samples were heated to 750 °C under 5% H_2_, held at this temperature for 4 h, and then cooled to room temperature. Afterward, the atmosphere was substituted with 10% O_2_ in N_2_, and the samples were reheated to 750 °C and held for 1 h. Finally, the samples were cooled to room temperature. Diffraction patterns were acquired every 40 s to monitor structural changes. Phase identification from selected patterns was conducted using MATCH! software, and Rietveld refinement was performed for phase quantification and cell parameter estimation with the FullProf Suite software. The Rietveld refinements were carried out on the raw diffraction data collected using the synchrotron radiation wavelength (λ = 0.4130 Å) without any wavelength conversion. After refinement, the diffraction patterns were converted to equivalent 2θ values corresponding to Cu Kα radiation (λ = 1.5406 Å) to enable comparison with standard reference patterns and literature data. All quantitative analyses and structural parameters were determined from the original synchrotron data.

In situ XPS measurements were conducted at the near-ambient pressure (NAP) branch of the CIRCE beamline of the ALBA Synchrotron, utilizing a PHOIBOS 150 NAP analyzer (SPECS GmbH). This system enables analyses under ultrahigh vacuum conditions but also allows for the collection of measurements with gas environments at pressures up to 20 mbar. The subsurface chemical composition of STF and STF-Ni was examined up to 550 °C under pure H_2_ and CO_2_ atmospheres in line with the photon energy set.^28^ High-resolution spectra were acquired for Sr 3d, Ti 2p, Fe 2p, Ni 2p, O 1s, and C 1s using a photon energy of 1000 eV in both atmospheres. The spectra were recorded with a pass energy of 10 eV and an energy step of 0.10 eV. The analyzed samples were prepared by pressing STF and STF-Ni powders into pellets (1 mm thickness and 1 cm diameter) and mounting them on a gold mesh support to prevent surface charging. Heating was achieved using an infrared laser system (808 nm), with temperature control realized with a K-type thermocouple in direct contact with the pellet’s surface. The total gas flow rate in the analysis chamber was maintained at 2 Ncc/min. The samples were heated under N_2_ at 10 K/min up to 550 °C. After a stabilization period of 30 min, the gas atmosphere was substituted with pure H_2_, and spectra were acquired after 1 and 2 h of exposure. Subsequently, the environment was changed to pure CO_2_, and after an equilibration period of 30 min, a set of measurements was collected once every hour over a period of 4 h. The spectral deconvolution was also performed using CasaXPS. A Shirley-type background subtraction was employed. The components of O 1s and Sr 3d were fitted with Gaussian–Lorentzian peaks (GL30). Complementary NEXAFS measurements were performed on the STF-Ni sample to investigate the oxidation states of Fe under the same experimental conditions studied with NAP-XPS. The spectra were acquired at the Fe L-edge (700–730 eV), and they were then normalized using Athena. In both the XRD and XPS in situ experiments, a gas purity of 5.5 grade (99.9995%) was chosen for the H_2_ and CO_2_ bottles, which guaranteed that the impact of residual impurities (e.g., O_2_, CO) was negligible. Although minor, humidity traces in these gases could not be excluded.

Thermogravimetric analyses (TGA) were performed on STF and STF-Ni powders using a Hitachi STA7300 TG-DTA system. For each experiment, a 50 mg sample was heated from room temperature to 750 °C at 10 K/min under a 5% H_2_ in He mixture. Once at 750 °C, a 120 min hold was performed. After this period, the atmosphere was substituted with pure CO_2_ while keeping the temperature constant for an additional 120 min. After exposure to CO_2_, the sample was cooled to room temperature at 15 K/min under He. Throughout the experiment, mass loss curves were continuously recorded. The surface area of the reduced powders after exposure to 5% H_2_ in Ar at 750 °C was also assessed via N_2_-BET analysis (TriStar Micromeritics 3000).

Finally, the STF and STF-Ni samples previously exposed to the 5% H_2_ in N_2_ mixture and to pure CO_2_ for 4 h at 750 °C were analyzed using transmission electron microscopy (TEM). For the preparation of TEM samples, a small quantity of perovskite powder was ultrasonicated in absolute ethanol and subsequently drop-cast onto a holey carbon copper grid. High-angle annular dark field scanning transmission electron microscopy (HAADF-STEM) and energy-dispersive X-ray spectroscopy (EDS) data were acquired using an FEI Tecnai G2 F20 microscope operated at 200  kV, equipped with an EDAX Super Ultrathin Window (SUTW) X-ray detector.

### 2.3 Cell Preparation and Electrochemical Characterization

The powders prepared via the SSS method were used to make fuel electrode inks by mixing with a vehicle (Heraeus V-737) on a 1:1.2 wt % ratio in a three-roll mill. Electrolyte-supported cells were prepared by screen-printing electrode inks onto a dense 150 μm-thick ScSZ electrolyte substrate (Fuel Cell Materials) as follows: first, two stacked gadolinium-doped ceria (GDC) barrier layers were screen-printed per side onto the electrolyte to avoid the formation of insulating layers on the cells. For the oxygen electrode, an LSCF-GDC composite ink (Fuel Cell Materials) was screen-printed using a circular cutout of 0.44 cm^2^ area. The main variation from cell to cell was the screen-printed fuel electrode, which included STF and STF-Ni with identical printed areas as the oxygen electrode. After printing a layer, it was dried at 150 °C for 1 h before firing. The firing temperature of STF, STF-Ni, and LSCF-GDC was 1100 °C, while the GDC barrier layers were fired at 1300 °C. Silver grids were also screen printed on the electrode surface using an Ag paste (Heraeus, T-23 GM) and dried at 150 °C for 1 h to realize the current collectors. The reference performance of each SOC was evaluated at 750 °C in 3% humidified hydrogen in SOFC mode right after the reduction of the fuel electrode. The reduction protocol consisted of heating the cells from room temperature to 750 °C at 1 K/min under a 3% humidified safety mixture (5% H_2_ in N_2_, 50 Ncc/min) supplied to the fuel side and air (50 Ncc/min) supplied to the oxygen side. Once at 750 °C, the hydrogen partial pressure was progressively increased 10% stepwise every 15 min until pure H_2_ (dry basis) was reached. The performance in reversible mode was collected afterward with a mixture containing 50% CO_2_/50% CO. Current/voltage (*I*/*V*) curves were measured between open-circuit voltage (OCV) and 0.35 V in SOFC mode and between 1.4 and 0.4 V in reversible mode.

## 3 Results

### 3.1 In Situ XRD Characterization

The evolution of the structures of STF and STF-Ni under reducing conditions was first investigated using in situ XRD during a thermal treatment in 5% H_2_ in N_2_. Figure 1 displays the XRD patterns obtained while heating to 750 °C. Both materials retained a cubic perovskite structure (*Pm–3m*), as confirmed by the indexing of the main reflection peaks (Figure S1 of the Supporting Information document). The refined unit cell parameters and the theoretical density of the main perovskite phase from representative XRD fingerprint conditions can be found in Tables S1 and S2. The peak shift to lower 2θ values with increasing temperature confirms progressive lattice expansion due to oxygen vacancy formation. The inclusion of Ni in the B-site for STF-Ni did not significantly alter the room-temperature cell lattice parameter (3.89 Å in STF vs 3.92 Å in STF-Ni), nor the peak positions relative to STF. Indeed, the main reflection peaks of all patterns were indexed to a cubic unit cell associated with Fe-doped strontium titanate (COD-1525550).

**Figure 1:**
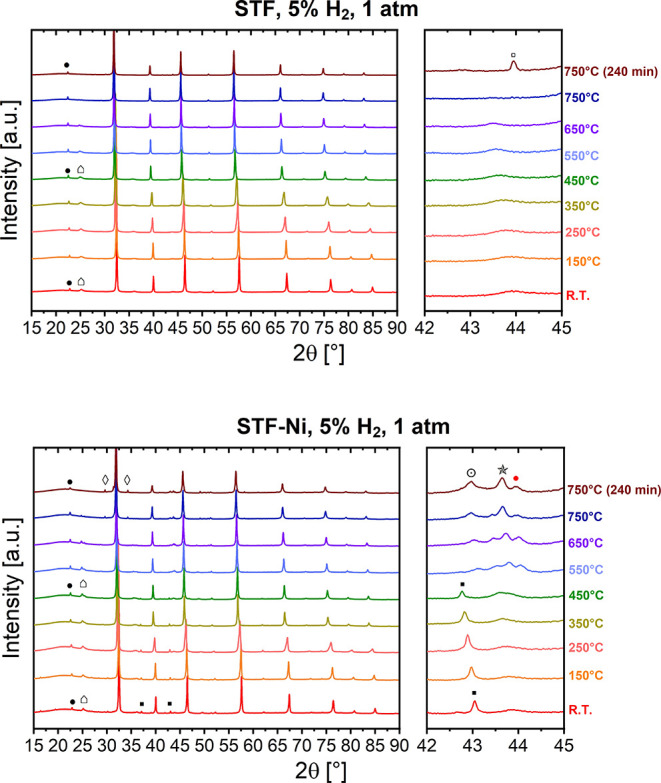
In situ XRD patterns of STF (top) and STF-Ni (bottom) measured while heating from room temperature to 750 °C in 5% H_2_ in an N_2_ mixture at atmospheric pressure. Symbols: (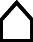) SrCO_3_, (■) NiO, (●) Ni–Fe alloy, (⊙) FeNi_3_ alloy, (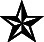) Ni, (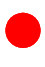) SrTiFeO_3_ phase, (◊) SrO, and (□) Fe.

The broad peak observed at room temperature with minimal intensity at ∼25° was ascribed to trace amounts of SrCO_3_ (∼2–3 wt %, Tables 1 and 2) coming from the exposure of the powder samples to atmospheric conditions before testing during handling and storage, evidencing their air sensitivity. Above 650 °C, this characteristic peak starts to attenuate due to thermal decomposition, and then it completely disappears at 750 °C.^29,30^ Other than the shift to lower angles, STF-Ni exhibited additional reflections: initial NiO peaks (∼43°) were present up to ∼450 °C, followed by the emergence of metallic Ni–Fe alloy peaks (43–44°, 49–50°), in line with nanoparticle exsolution.^20^ The rapid transition from NiO to alloyed phases suggests that Sr substoichiometry and Ni doping play a catalytic role in facilitating Fe reduction and exsolution. The minor peak visible between 51° and 52° in both samples was attributed to the (210) perovskite reflection, which shifted with temperature, as expected. At 750 °C, STF-Ni started presenting the formation of peaks at 29° and 34°, which were related to the formation of minimal amounts of SrO. The decomposition of SrCO_3_ into SrO under CO_2_-lean reducing conditions is a plausible source for the SrO in STF-Ni. However, our comparative experiments suggest that it is not the only origin. In STF, the SrCO_3_ traces present at room temperature disappear by 750 °C without the appearance of SrO peaks. This indicates that the released Sr is reincorporated into the lattice or remains in a disordered surface layer below XRD detection, coherently with the SrCO_3_ ⇌ SrO + CO_2_ equilibrium at low pCO_2_. In contrast, STF-Ni shows a clear SrO reflection persistence under the same conditions, which cannot be explained solely by the small carbonate fraction present at room temperature. The key difference is the occurrence of Fe–Ni exsolution, which increases the oxygen vacancy concentration and modifies the near-surface structure of the perovskite, thereby driving additional Sr segregation and SrO formation. This formation may be enhanced by the exsolution of Ni and Fe, which do not reincorporate into the bulk, thereby hindering the reintegration of Sr into the lattice. As a result, Sr remains segregated and readily forms SrO, which is thermodynamically stable under 5% H_2_, since Sr^2+^ is already in its lowest oxidation state and cannot be further reduced.^31^

**Table 1:** Phase Fractions Calculated for the XRD Patterns Collected on the STF Samples during the Experiments for Reducing (5% H_2_) and Oxidizing Conditions (10% O_2_)

T [°C]	Mixture	STF [%]	SrCO_3_ [%]	Fe [%]
25	5% H_2_ in N_2_	98.19	1.81	0
550		97.96	2.04	0
750 (0 min)		98.99	1.01	0
750 (240 min)		98.58	0	1.42
25	10% O_2_ in N_2_	99.54	0	0.46
550		99.98	0	0.02
750 (0 min)		100	0	0
750 (64 min)		100	0	0

**Table 2:** Phase Fractions Calculated for the XRD Patterns Collected on the STF-Ni Samples during the Experiments for Reducing (5% H_2_) and Oxidizing Conditions (10% O_2_)

T [°C]	Mixture	STF [%]	SrCO_3_ [%]	NiO [%]	FeNi [%]	FeNi_3_ [%]	Ni [%]	SrO [%]
25	5% H_2_ in N_2_	97.20	1.59	1.16	0	0	0	0
750 (0 min)		93.20	2.95	0	1.48	1.29	1.07	0
750 (240 min)		94.58	0	0	1.76	0.16	2.02	1.48
25	10% O_2_ in N_2_	96.61	0	0	0.97	0.64	1.79	0
750 (0 min)		96.25	1.40	2.20	0	0	0.15	0
750 (64 min)		96.37	0	2.69	0	0	0	0.94

Figure S2 captures the time-resolved patterns during a 4-h dwell at 750 °C in 5% H_2_, with the final fingerprint at minute 240 being reported also in Figure 1. STF presented a pattern with minimal variation, aside from a reflection peak of small intensity, which appeared at 44° after 30 min of dwell. This peak intensified with the dwell time, suggesting gradual formation of a secondary phase eased by the loss of lattice oxygen. The position and evolution of this peak were consistent with the (110) reflection of metallic α-Fe, indicating its migration from the perovskite lattice. The phase quantification (Table 1) revealed a concentration of up to 1.42 wt % of metallic Fe, with the perovskite phase remaining dominant at 98.6 wt % after 240 min. In contrast, the exsolution process in STF-Ni appears to be highly complex and involves the formation and transformation of different crystalline phases, including intermediate metallic Ni- and Fe-based ones. This complexity is expected given the A-site deficiency of the parent oxide with respect to stoichiometric STF and given the presence of highly reducible Ni cations at the B site. Figure 1 shows that metallic phases start exsolving from the host lattice at 450 °C, when the series of peaks around 44° becomes visible, and the intensity of the NiO peak begins to decrease. At 550 °C, NiO is completely reduced, and the corresponding peak disappears. Going from 550 to 750 °C, three distinct metallic phases, namely, FeNi, FeNi_3_ and Ni evolve and modify. The quantitative analysis (Table 2) shows that after 240 min at 750 °C, STF-Ni contained 1.76 wt % FeNi, 0.16 wt % FeNi_3_, and 2.02 wt % elemental Ni, which corresponded to a total of ∼4 wt % metallic phase content. The presence of both FeNi and FeNi_3_ indicates that a range of alloy compositions forms under these conditions, with FeNi forming preferentially, as already reported by Zhu et al.^14^ However, to the best of our knowledge, this is the first time such behavior has been reported with temperature resolution, enabled by the high sensitivity of synchrotron radiation. Alloy-related peaks were already visible during the heat-up stage (Figure 1) and continued to intensify during the dwell. The quantitative analysis (Table 2) shows that after 240 min at 750 °C, STF-Ni contained 1.76 wt % FeNi, 0.16 wt % FeNi_3_, and 2.02 wt % elemental Ni, which corresponded to a total of approximately 4% metallic phase content. The presence of both FeNi and FeNi_3_ phases indicates that a range of alloy compositions forms under these conditions, with FeNi forming preferentially. To the best of our knowledge, this is the first time such behavior has been reported, enabled by the high sensitivity of synchrotron radiation. No reflections related to Ruddlesden–Popper or to other secondary perovskite phases were detected during either the heat-up or dwell stages, which suggested that the primary mode of structural change is the selective formation of metallic nanoparticles (STF-Ni) or metallic Fe (STF) rather than phase decomposition up to 750 °C.

To investigate the reversibility of the redox processes and the structural stability of the reduced perovskites, both STF and STF-Ni were subjected to reoxidation. After the 4 h reduction in 5% H_2_, the samples were exposed to an oxidizing atmosphere consisting of 10% O_2_ in N_2_. This second XRD experiment involved heating the previously reduced samples to 750 °C, followed by an isothermal dwell of 64 min. This experiment aimed to evaluate the extent to which the exsolved metallic species could be reincorporated into the perovskite lattice and to assess whether secondary phases, such as SrO or NiO, persisted or were eliminated under oxidizing conditions. As shown in Figure 2, both STF and STF-Ni retained their cubic perovskite symmetry throughout the reoxidation process. Subtle shifts of the main reflections to lower 2θ values were observed as the temperature increased, reflecting thermal lattice expansion similar to that seen during reduction. For STF, the peak at ∼44° previously attributed to metallic α-Fe after reduction progressively weakened and ultimately disappeared at 750 °C. This indicated the complete reincorporation of Fe into the perovskite lattice, without formation of FeO_*x*_ intermediate phases. The phase quantification (Table 1) supports this observation: while 0.46 wt % Fe was still detectable at room temperature after the switch to 10% O_2_, it dropped to 0.02 wt % at 550 °C and was no longer detectable at 750 °C, demonstrating the full reversibility of Fe reduction in STF. In contrast, for STF-Ni, as the temperature rose during reoxidation, peaks corresponding to the Ni–Fe alloy decreased in intensity, which suggested that some dissolution or transformation of the alloyed phase took place. However, instead of fully reincorporating into the lattice, the reoxidized Ni primarily reappeared as NiO, as evident from the emergence and growth of a broad peak centered at ∼43° at 550 °C. Moreover, during the heating ramp, very small peaks appeared around 35°, which could be attributed to Fe_3_O_4_ species (Figure 2). This observation suggests that the metallic Fe present in the Ni–Fe alloy is initially oxidized to form Fe_3_O_4_ on the surface of the host perovskite.^25^ Upon further heating, these oxide species progressively redissolve into the perovskite lattice, leading to their complete incorporation at 750 °C. The peaks at 29° and 34° almost instantaneously disappeared after exposure at room temperature to 10% O_2_, which indicates that under these conditions, SrO likely reacts with residual Fe and Ti species to reintegrate into the perovskite structure. Overall, the main diffraction peaks remain largely unchanged across the temperature range, indicating that the primary cubic crystal structure of both STF and STF-Ni is stable under these conditions.

**Figure 2:**
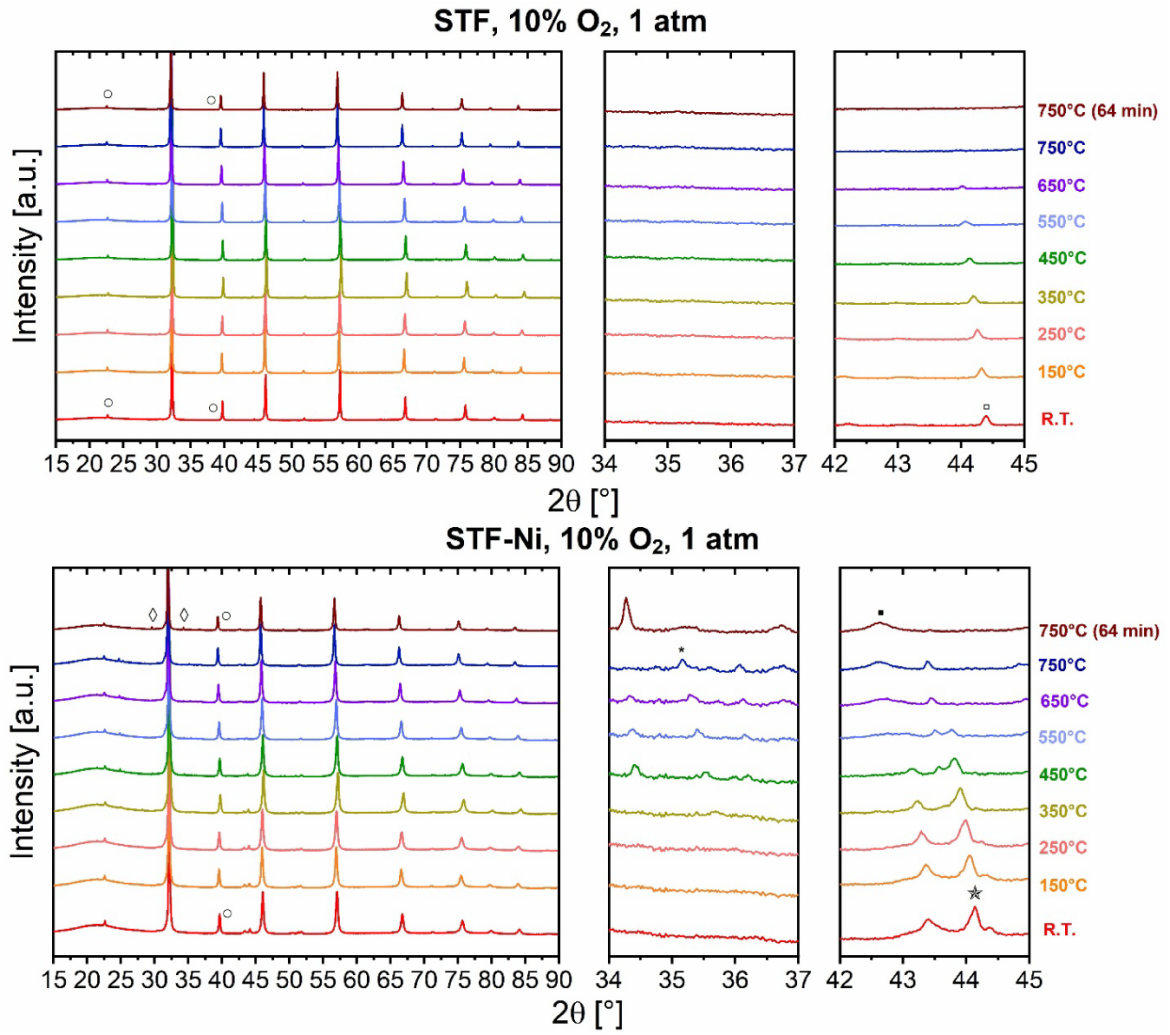
In situ XRD patterns of STF (top) and STF-Ni (bottom) measured while heating from room temperature to 750 °C in 10% O_2_ in an N_2_ mixture at atmospheric pressure. Symbols: (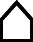) SrCO_3_, (■) NiO, (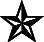) Ni–Fe alloy, (○) SrTiFeO_3_ phase, (◊) SrO, (□) Fe, and (*) Fe_3_O_4_.

The time-resolved XRD patterns during the dwell of 64 min in 10% O_2_ at 750 °C are reported in Figure S3, whereas the final fingerprint at minute 64 is reported in Figure 2. STF presents no pattern variation within the studied period. In contrast, STF-Ni exhibits a more dynamic structural behavior during the dwell. The peaks of SrO reappeared from minute 32 onward and remained unaltered until the end of the experiment. Additionally, the quantitative phase analysis indicates that the NiO content increased from 2.20 wt % at the start of the dwell (0 min in Table 2) to 2.69 wt % after 64 min, while the other metallic phases were no longer detected. This indicates that, upon reoxidation, Ni tends to stabilize as NiO, most likely as surface-bound particles. Notably, the amount of NiO found after the oxidation was larger than the amount observed on the pristine STF-Ni sample, which strongly suggests that part of the exsolved Ni, either from the metallic state or in the alloyed nanoparticle, did not reincorporate into the perovskite lattice. These observations confirm the incomplete reversibility of the exsolution process in STF-Ni, where Ni oxidation leads to phase separation rather than reintegration and correlates with the persistence of SrO reflections during reoxidation. This behavior, not observed for STF, can be allegedly associated with the higher reactivity of STF-Ni and with the incomplete reincorporation of the exsolved metals, leaving SrO segregated at the surface. Once present, such segregated SrO is known to be highly reactive and may readily form carbonates upon exposure to even trace amounts of CO_2_.

A comparative summary of the XRD patterns for STF and STF-Ni at room temperature under pristine, reduced (5% H_2_), and reoxidized (10% O_2_) conditions is provided in Figure S4. This review emphasizes the structural modifications observed during the in situ sequences and highlights the differing redox responses of the two materials. For STF, only minor shifts in the diffraction peaks are visible upon reduction, which are due to lattice expansion from oxygen vacancy formation. The transient appearance of a peak at ∼44°, associated with metallic Fe, is no longer present in the reoxidized sample, confirming its full reintegration into the perovskite lattice and the high degree of redox reversibility. In contrast, STF-Ni displays a more complex structural evolution. The reduced sample shows distinct additional reflections from Ni–Fe alloy formation and SrO segregation, in agreement with Figure 2. Upon reoxidation, even if the alloy peaks diminish, they are replaced by a broad reflection attributed to NiO, indicating incomplete reintegration of Ni into the lattice. Moreover, peaks assigned to SrO remain visible, suggesting that A-site cation segregation is only partially reversible.

### 3.2 In Situ NAP-XPS Characterization

The redox behavior of STF and STF-Ni was investigated with in situ XPS. In these experiments, STF and STF-Ni samples were analyzed under reducing and oxidizing conditions at 550 °C in pure H_2_ and pure CO_2_, respectively. Since the results of in situ XRD (Figure 1) showed that exsolution in STF-Ni initiates as early as 450 °C, 550 °C was deemed sufficient to activate the surface redox processes of interest, while the use of pure atmospheres further enhanced the chemical driving force for surface transformations. Moreover, in contrast to the XRD experiments, wherein O_2_ was used as the oxidizing agent, CO_2_ was selected for the NAP-XPS studies to directly investigate the surface reactivity of the materials toward carbon-containing oxidants. This choice is especially relevant in the context of SOC’s operations in CO_2_ electrolysis or H_2_O/CO_2_ coelectrolysis, where exposure to CO_2_ can lead to performance degradation.^32^

The analysis focused on the Sr 3d, O 1s, Fe 2p, and Ni 2p core levels. The spectrum of Sr 3d was deconvoluted with two main components: a low-binding-energy component assigned to the perovskite lattice and a higher-binding-energy component assigned to the surface. Since the Sr 3d spectra showed spin–orbit splitting, the doublets Sr 3d_3/2_ and Sr 3d_5/2_ peaks were used for both lattice and surface components. The surface doublet was associated with the presence of SrO, SrCO_3_, or Sr­(OH)_2_ species.^33,34^ The lattice component was associated with Sr present in the perovskite bulk. For the O 1s spectrum, three singlets were used for the deconvolution: a singlet corresponding to oxygen within the perovskite lattice in the lower-binding-energy region and two additional surface singlets. Peaks labeled as other oxides represented oxygen species such as OH^–^ or O^2–^ bound to metal ions (from Sr or transition metals) in the perovskite’s surface. The remaining surface peak was attributed to chemisorbed oxygen species, which strongly varied according to the state of surface oxygen during reduction in pure H_2_ and oxidation in CO_2_. The Fe 2p and Ni 2p spectra were not deconvoluted. Nonetheless, indications of the change in oxidation state toward the metallic form (Fe^0^, Ni^0^) are provided.

Binding energy shifts are observed in STF and STF-Ni for the Sr 3d and O 1s high-resolution spectra during reduction (Figures 3 and 4), due to the changes in the Fermi level induced by variations in the atmosphere’s oxygen partial pressure.^35,36^ Upon exposure to H_2_, the formation of oxygen vacancies lowers the chemical potential of oxygen and shifts the Fermi level downward with respect to the vacuum level, leading to an increase in the binding energy. Unlike Sr 3d and O 1s, the Ni 2p and Fe 2p spectra exhibited a less pronounced shift due to the formation of phases with lower oxidation states, such as metallic Fe and Ni, which introduced new spectral features and displacement to smaller binding energies. The additional peak at 536.5 eV in the O 1s spectrum (Figures 3F and 4F) was assigned to gas-phase CO_2_. Literature evidence^37^ confirms that free CO_2_ molecules exhibit an O 1s peak at ∼536.3–536.5 eV, which distinguishes them from chemisorbed CO_2_ or carbonate species, which instead appear at lower binding energies (∼531–532 eV).

**Figure 3:**
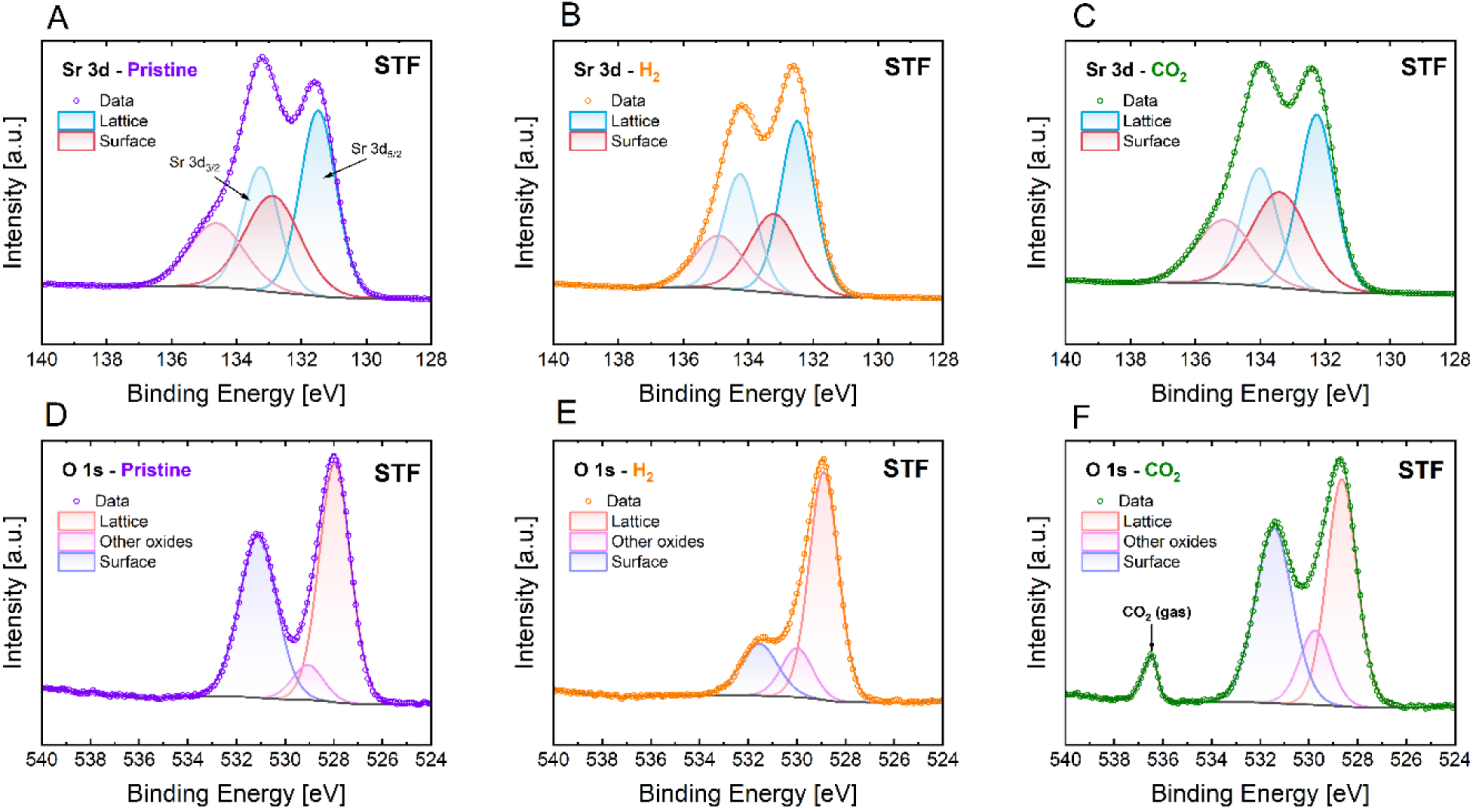
In situ NAP-XPS spectra of Sr 3d (A–C) and O 1s (D–F) core levels for the STF sample under different conditions: pristine (A, D), after H_2_ exposure (B, E), and after CO_2_ exposure (C, F).

**Figure 4:**
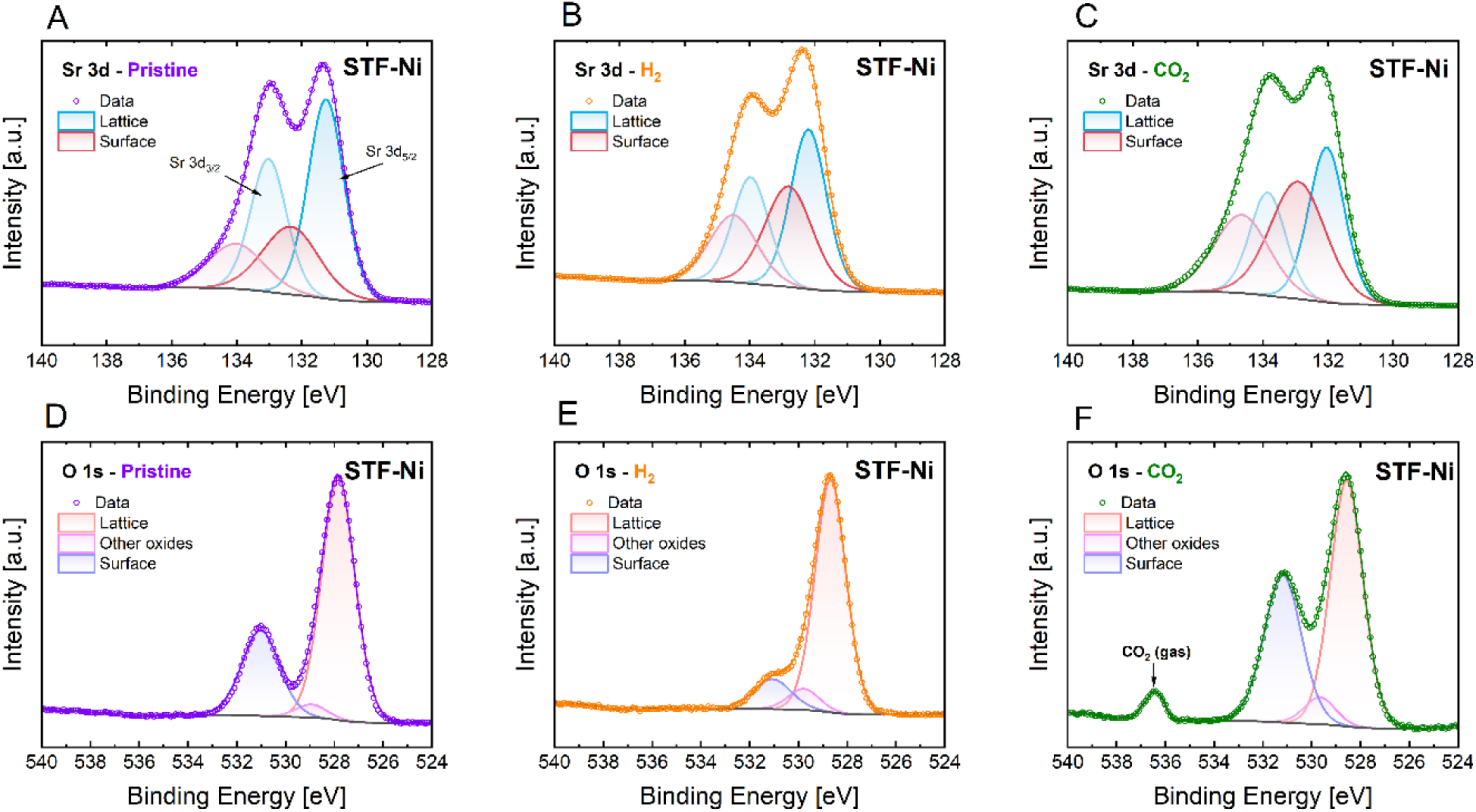
In situ NAP-XPS spectra of Sr 3d (A–C) and O 1s (D–F) core levels for the STF-Ni sample under different conditions: pristine (A, D), after H_2_ exposure (B, E), and after CO_2_ exposure (C, F).

The spectra of STF reveal significant surface changes under different gas environments in the Sr 3d and O 1s core levels (Figure 3). In the pristine state, the Sr 3d spectra show well-defined Sr 3d_5/2_ and Sr 3d_3/2_ doublets. The broader surface peak in the 3d 3/2 position, which appears almost as a shoulder, might be related to the presence of SrCO_3_ in the sample, coming from the pellet preparation process and exposure to ambient conditions. Upon exposure to H_2_, the surface Sr contribution decreases, indicating partial reduction or surface reconstruction, even if the bulk remains largely unaffected.^38^ Upon exposure to CO_2_, the surface Sr component increases due to carbonate formation, as supported by the O 1s spectra. The O 1s spectrum in the pristine state exhibits an almost 1:1 area ratio between the bulk and surface contributions (Table 3). After exposure to H_2_, the surface oxygen contribution decreases, which suggests oxygen loss and vacancy formation. In contrast, exposure to CO_2_ leads to a noticeable increase in surface oxygen. A similar behavior is observed in STF-Ni (Figure 4), though with minor differences. In the pristine state, STF-Ni has a smaller Sr surface contribution compared to STF. Upon exposure to H_2_, the surface contribution from Sr 3d increases, indicating an Sr surface enrichment. This suggests that in STF-Ni, Sr migration to the surface is enhanced, likely due to Ni, which modifies the defect chemistry and the oxygen vacancy formation. The formation of oxygen vacancies in a reducing atmosphere, which readily occurs in STF-Ni, may promote Sr mobility and surface reconstruction, leading to a higher Sr surface-to-bulk ratio compared to the pristine state. The O 1s spectra show the depletion of surface oxygen species, confirming the formation of oxygen vacancies under H_2_ for STF-Ni. When exposed to CO_2_, the contribution of the peak related to surface Sr (∼133 eV) further increases, indicating the formation of strontium carbonate via the reaction between gas-phase CO_2_ and surface Sr-containing species, likely SrO.

**Table 3:** Comparison of the Relative Percentage and Ratio of Deconvoluted Components for the O 1s Spectra and for the Sr 3d Spectra^a^

	Condition								
	Pristine			H_2_			CO_2_		
Sample	Surface	Bulk	Ratio	Surface	Bulk	Ratio	Surface	Bulk	Ratio
**STF (O 1s)**	49%	51%	0.95	34%	66%	0.52	56%	44%	1.29
**STF-Ni (O 1s)**	31%	69%	0.45	20%	80%	0.25	44%	56%	0.78
**STF (Sr 3d)**	46%	54%	0.86	15%	85%	0.18	38%	62%	0.63
**STF-Ni (Sr 3d)**	25%	75%	0.33	15%	85%	0.18	30%	70%	0.42

a The contributions from the surface and from other oxides were lumped in the surface term reported in the table. In the case of Sr spectra, calculations are done considering the sum of each peak of the doublet.

Right from the pristine state, STF-Ni presented almost half of the O 1s surface/bulk ratio of STF, indicating that a partial reduction took place while heating in N_2_ under vacuum. In both STF and STF-Ni, the surface/bulk ratio decreases from the pristine to the reduced condition due to the formation of oxygen vacancies and the loss of surface oxygen (Table 3). However, STF-Ni shows a smaller ratio than STF (0.25 vs 0.52), which suggests that the presence of Ni either enhances bulk oxygen retention or facilitates surface oxygen removal. On exposure to CO_2_, the surface/bulk ratio increases for both materials with respect to the reduced condition, suggesting oxygen uptake or carbonate formation preferentially at the surface. STF shows a higher ratio under CO_2_ when compared to STF-Ni (1.29 vs 0.78), which suggests a stronger interaction between CO_2_ and the surface oxygen component.

The quantification of the Sr 3d spectra was performed by separating the surface-related Sr species from the contribution of the bulk perovskite and evaluating their surface/bulk ratios. In the pristine state, STF exhibits a higher surface-associated Sr contribution (46%, 0.86 ratio) compared with STF-Ni (25%, 0.33 ratio). This difference likely arises from a combination of Sr surface enrichment and a more pronounced SrCO_3_ surface component in STF at ambient conditions. Under H_2_, both materials show a pronounced decrease in the surface-related Sr signal, with surface/bulk ratios decreasing to an equal level (0.18). This behavior is consistent with the reductive decomposition and removal of surface carbonate species originally formed during the samples’ storage, which are destabilized under reducing conditions and desorb from the surface. This interpretation is further supported by the XRD measurements performed in 5% H_2_ (Figure 1), where the reflection associated with SrCO_3_ progressively disappears at around 550 °C. Upon exposure to pure CO_2_, both materials exhibited a marked increase in the surface Sr contribution, with higher surface/bulk ratios (0.63 for STF and 0.42 for STF-Ni). As shown by Jung and Tuller,^39^ the smaller amount of superficial Sr in STF-Ni than in STF observed on the pristine samples (Table 3) is explained by the 3% Sr deficiency of STF-Ni, which is instead absent in Sr-stoichiometric STF. These authors have in fact shown that Sr segregation can be limited by introducing A-site deficiency, due to the mitigation of mechanical strain lattice gradients. Hence, Sr deficiency rather than Ni doping explains the lower fraction of surface Sr found in STF-Ni in its initial state.

The Fe 2p spectra reveal differences in the redox behavior of Fe in STF (Figure 5A) compared to that in STF-Ni (Figure 5B), highlighting the influence of Ni on Fe oxidation states. In the pristine samples, Fe in both STF and STF-Ni exhibits predominantly an Fe^3+^/Fe^2+^ mixed oxidation state. However, when the sample is exposed to H_2_, the response differs between the two materials. In STF, Fe undergoes partial reduction, confirmed by shifts in the binding energy and by changes in peak intensity, but remains within the Fe^3+^/Fe^2+^ oxidation states without a full reduction to the metallic state. In contrast, Fe experiences a significantly stronger reduction in STF-Ni, with the appearance of metallic Fe^0^, a feature that is absent in STF. Lower binding energies in the Fe 2p 3/2 and 1/2 peaks of STF-Ni might also suggest that the oxidation state is closer to 2+ compared to STF. This observation implies that Ni enhances Fe reducibility under H_2_. When exposed to CO_2_, the oxidation state of Fe remains largely unchanged for STF, with Fe maintaining its Fe^3+^/Fe^2+^ state (Figure 5A). However, in the case of STF-Ni, the absence of Fe^0^ formation under CO_2_ (Figure 5B) indicates the possible reabsorption of Fe into the perovskite, even under weak oxidative conditions.

**Figure 5:**
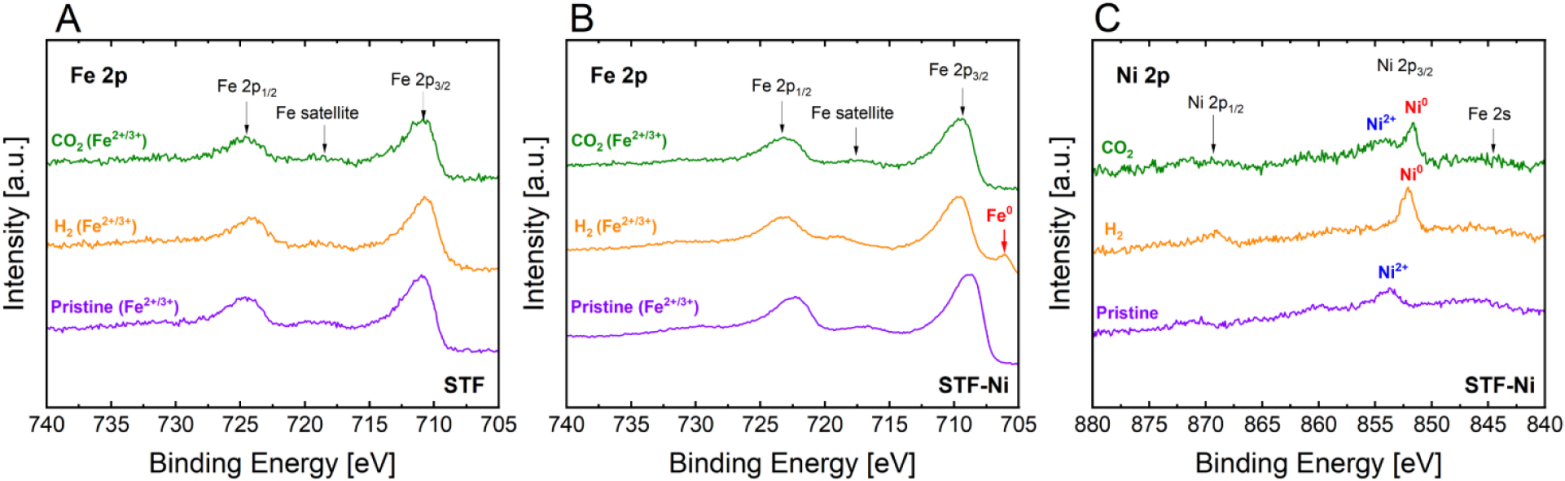
In situ NAP-XPS spectra under different conditions (pristine, after H_2_ exposure, and after CO_2_ exposure): (A) Fe 2p core level for STF, (B) Fe 2p core level for STF-Ni, and (C) Ni 2p core level for STF-Ni.

The Ni 2p spectra in STF-Ni (Figure 5C) also reveal changes in the Ni oxidation states under different gas environments. In the pristine state, Ni is primarily present as Ni^2+^. A notable shift occurs in H_2_, with the appearance of metallic Ni^0^ at lower binding energies and the disappearance of the Ni^2+^ shoulder, which confirms that Ni reduces completely. The presence of Ni^0^ also suggests that Ni is not fully stabilized in the perovskite lattice and it is prone to exsolve under low oxygen partial pressure. Metallic Fe^0^ in the Fe 2p spectra of STF-Ni further supports this, confirming that the reduction of Ni facilitates the concurrent reduction of Fe. Even if the NAP-XPS spectra were not collected at 750 °C due to limitations in the laser heating system, significant distinctions were yet observed at 550 °C: in agreement with the XRD patterns (Figure 1), no Fe^0^ peak was detected on STF at 550 °C, corroborating the observation that metal Fe particles segregate only at much higher temperature (750 °C) and that Ni instead accelerates the reduction and exsolution of Fe in STF-Ni. In CO_2_, Ni undergoes partial reoxidation, with an increase in the contribution from Ni^2+^. This result suggests that even weak oxidizing conditions promote the formation of Ni passivation layers, which allow for keeping part of the metallic Ni at the near-surface level. Differently, complete readsorption of the reduced species is observed in the case of Fe. Stronger oxygen partial pressures in the feed may be required to fully reintegrate Ni into the perovskite and ensure clean surface regeneration. Overall, the combination of Fe and Ni redox dynamics suggests that Ni not only enhances Fe reducibility under H_2_, but also exhibits its own redox transitions, resulting in modifications of the electrocatalytic activity of STF-Ni (Section 3.5). Under H_2_, appreciable improvements in the fuel electrode performance might become more evident for STF-Ni as metallic Fe and Ni enhance the electronic conductivity of the perovskite. Under CO_2_, improvement related to the presence of Ni in the formulation might become less apparent since Fe, which is the quantitatively predominant phase in the formulation, exhibits higher oxidation states, which are closer to those of STF.

In terms of Fe oxidation states, Figure 6 provides further insight into the redox response of the surface of STF-Ni under reducing and oxidizing conditions as measured with NAP-NEXAFS. When comparing the spectra collected under H_2_ and CO_2_ (panel A) to the reference (panel B), the spectrum acquired under CO_2_ closely resembles that of Fe^3+^, with sharper L_3_ and L_2_ peaks centered at ∼710 eV and ∼722 eV, respectively. In contrast, under H_2_, the L_3_ peak becomes broader, while the L_2_ feature diminishes in intensity, which indicates a partial reduction toward Fe^2+^. Moreover, although the appearance of an additional shoulder at lower photon energies in the L_3_ region could suggest the formation of metallic Fe^0^, the XPS data (Figure 5B) show that the majority of Fe remains in the Fe^2+/^Fe^3+^ oxidation states, and that no Fe^0^ is present in the Fe 2p spectra. Therefore, the spectral variation observed in CO_2_ can be more reasonably attributed to a redistribution within the Fe^2+/^Fe^3+^ redox couple, with a predominance of the relative amount of Fe^3+^. Minor imprecision in the spectra calibration could have led to a slight misalignment of the signals.

**Figure 6:**
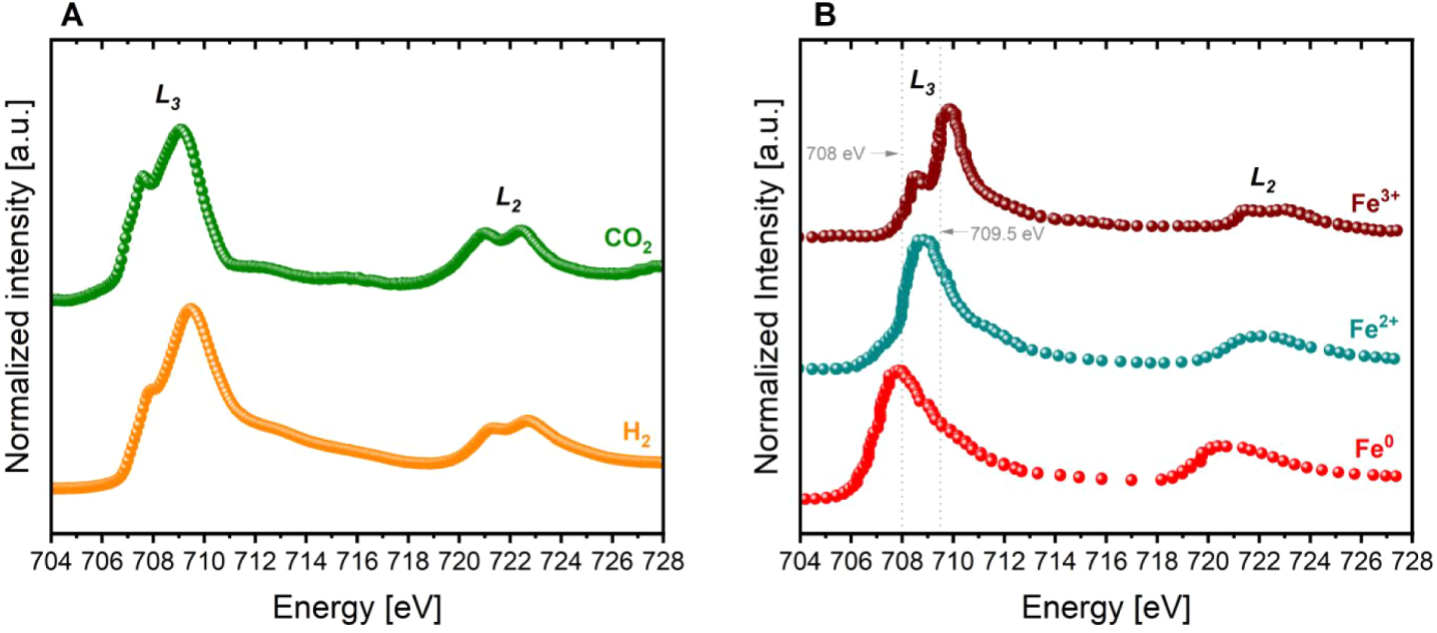
Fe L-edge NAP-NEXAFS (A) TEY signal measured for STF-Ni in reduced (H_2_) and reoxidized conditions (CO_2_). (B) Reference spectra of Fe0, FeO (Fe^2+^), and Fe_2_O_3_ (Fe^3+^).

### 3.3 Thermogravimetric Experiments

Figure 7 displays the results of the TGA analyses performed on STF and STF-Ni. Initially, both samples underwent weight loss during heating in a 5% H_2_/He atmosphere. The weight loss reached approximately 8% for STF and 11% for STF-Ni from room temperature to 750 °C and began as early as 50 °C in both samples. However, temperature-programmed reduction experiments carried out by our group under analogous conditions (5% H_2_ in Ar, 10 K/min heating rate) showed that the onset of H_2_ uptake occurs above 300 °C for both STF and STF-Ni.^27^ Considering that the samples were not pretreated prior to utilization in TGA, it is reasonable to assume that the desorption of humidity contributed to the initial weight loss (2.5 wt % for STF and 4.8 wt % for STF-Ni) before reaching 300 °C.^28^ The more rapid weight loss observed for STF-Ni indicates a higher reduction rate compared to STF, which would be due to the presence of Ni, as supported by the results of NAP-XPS. Such slower reduction rate of STF compared to STF-Ni also agrees with the fact that, in STF, metallic Fe is observed only at 750 °C, whereas the first traces of the Ni–Fe phase appear in STF-Ni as early as 450 °C. During the 120 min hold at 750 °C, both STF and STF-Ni kept on reducing with additional weight losses of 0.48 and 1.29 wt %, respectively. Such continuous reduction indicates that the presence of Fe plays a critical role in the stability of the fuel electrode. The absence of stabilization in 5% H_2_ also suggests that minimal oxygen partial pressure in the gas supply could induce structural instability during SOC operations, especially in STF-Ni, which exhibits a more dynamic redox response. As soon as the supply was substituted by pure CO_2_, an almost instantaneous weight gain was observed for both STF and STF-Ni, in line with the strong interactions with the gas phase found in NAP-XPS. The extent of this weight recovery is more pronounced in STF-Ni (+5.9 wt %) than in STF (+2.6 wt %). This behavior can be attributed to the higher oxygen vacancy concentration generated during the reduction step, which increases the perovskite’s ability to adsorb CO_2_. Furthermore, unlike the reduction step, a plateau was reached within 90 min of exposure to CO_2_ as, possibly, the reoxidation and the carbonate formation processes reached an equilibrium within this time frame.

**Figure 7:**
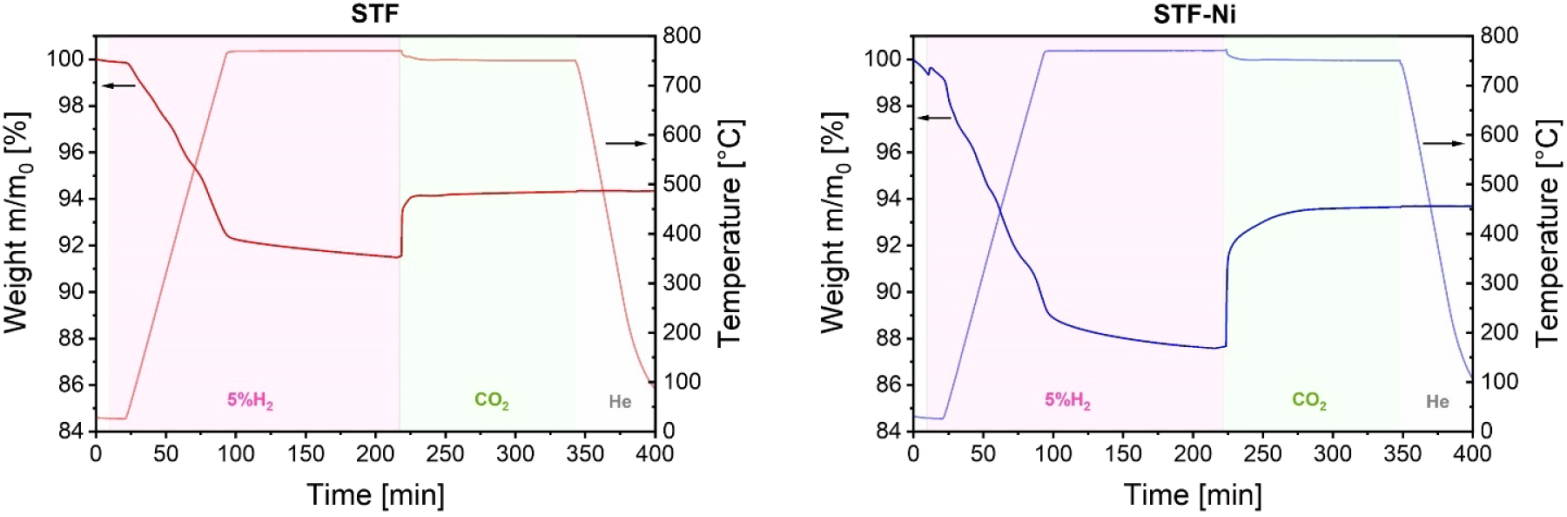
TGA analysis was performed on STF and STF-Ni powder samples. The samples were heated from room temperature to 750 °C under 5% H_2_ in He, followed by a 120 min dwell at 750 °C. The H_2_/He mixture was then substituted with CO_2_ while maintaining the temperature for an additional 120 min. The samples were cooled to room temperature under He.

### 3.4 TEM Characterization

TEM images were collected on STF and STF-Ni powders under three different conditions: pristine, reduced in 5% H_2_, and after exposure to pure CO_2_. In their pristine state, both materials show a compact and relatively homogeneous structure (Figure 8A and B). After reduction, exsolution occurred in STF-Ni, leading to the formation of nanoparticles with an average diameter d_p_ of 15 nm (Figure 8C). The surface of STF remained mostly unchanged compared to its pristine state, with the appearance of a few Fe nanoparticles and some larger Fe aggregates (Figure 5S). This picture agrees with the process of Fe segregation observed by Santaya et al.^40^ after the reduction of STF, which leads to the formation of Fe aggregates with a nonhomogeneous size distribution and of platelets located at the grain boundaries. Such a process contrasts with the ordered and homogeneous exsolution of Ni–Fe nanoparticles, which takes place on STF-Ni. After exposure to CO_2_, a structural transformation is evident in STF-Ni, as the nanoparticles grew in size (25 nm d_p_). EDS line scans were applied to analyze the composition of the nanoparticles, and the compositional maps derived are reported in Figure S6. After reduction (Figure 8D), the elemental distribution indicates that Ni and Fe migrated toward the surface, forming metallic alloyed Ni–Fe nanoparticles, while Sr and Ti remained in the perovskite matrix. The line scan after CO_2_ exposure (Figure 8E) shows that Fe reabsorbed into the perovskite bulk, whereas Ni-rich nanoparticles remained at the surface. This compositional shift confirms that Fe has a stronger tendency to reincorporate into the lattice, even after exposure to a weaker oxidant such as CO_2_ with respect to O_2_ or air, while Ni preferentially remains on the surface. On the other side, TEM images of STF from our previous work^27^ reveal significant Sr segregation after exposure to CO_2_, with the appearance of a film primarily composed of Sr, C, and O. The exclusive presence of these elements strongly suggests the formation of SrCO_3_, due to the reaction between segregated Sr and CO_2_ from the gas phase. This observation agrees with the in situ XPS results (Section 3.2), and further supports the assumption that Sr readily migrates to the surface. For STF-Ni, complementary TEM-EDS analyses (Figure S6 and elemental analyses of Figure S7) indicate that Sr segregation is also present but occurs only in certain regions of the powders, with a nonhomogeneous distribution. In turn, such surface modifications impact the SOC’s performance of STF and STF-Ni, as a carbonate insulating layer strongly hinders the electrocatalytic activity.

**Figure 8:**
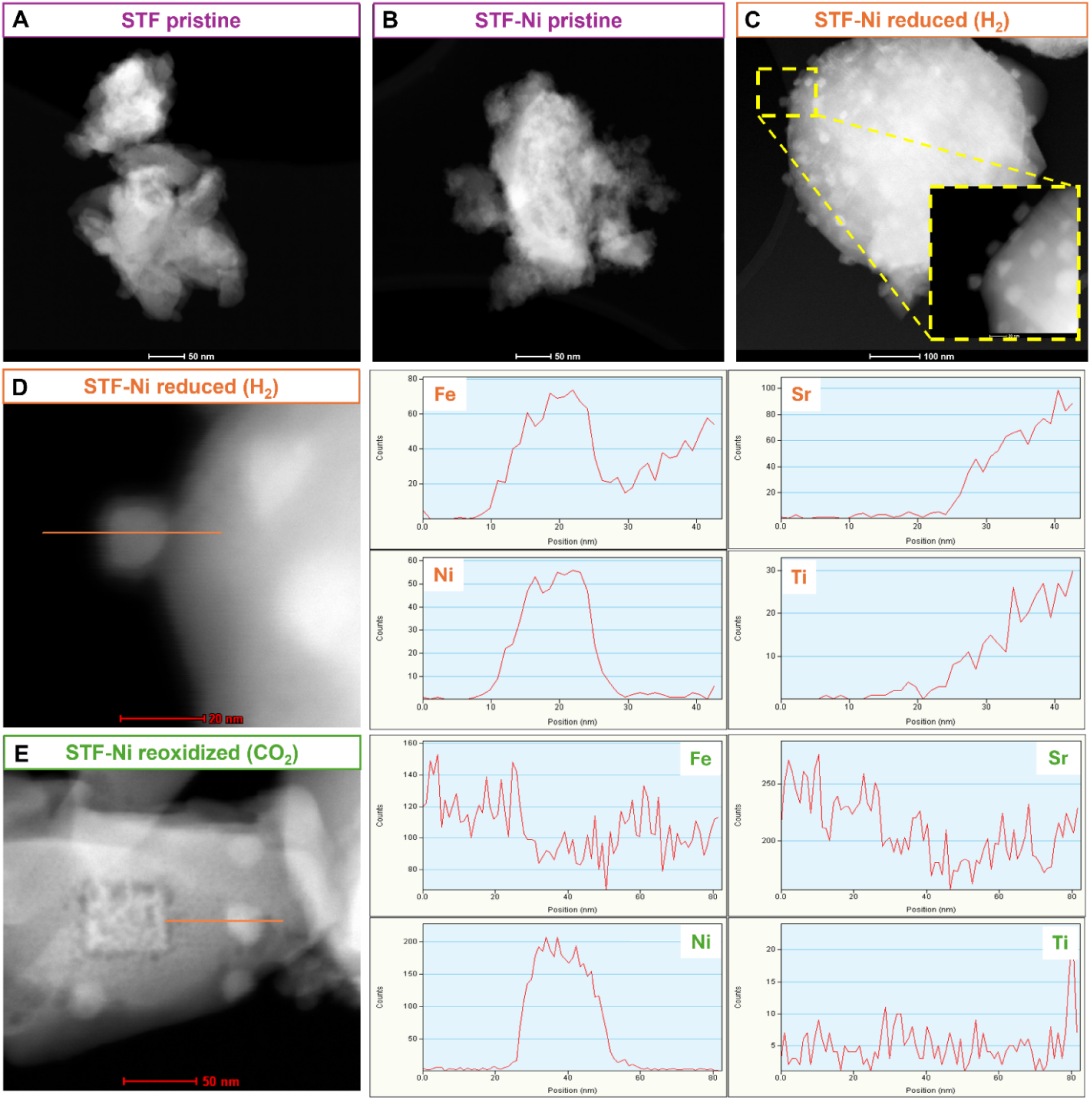
TEM images of pristine (as-prepared) STF (A) and STF-Ni (B) powders. (C) TEM image of STF-Ni after reduction in 5% H_2_, showing evidence of exsolution (highlighted in the inset). (D) TEM image and corresponding EDS line scans of Fe, Sr, Ni, and Ti across an exsolved nanoparticle in reduced STF-Ni. (E) TEM image and EDS line scans of the STF-Ni nanoparticles after reoxidation in CO_2_, illustrating changes in the elemental distribution.

### 3.5 Electrochemical Characterization

Figure 9A displays the performance of the SOCs in the fuel cell mode with 3% humidified H_2_ and air. Compared to the STF-based cell, the STF-Ni cell reached higher maximum current density at 0.35 V (1092 vs 907 mA/cm^2^), and larger maximum power density at 0.5 V (434 vs 357 mW/cm^2^). Under reversible conduction with a mixture of 50% CO and 50% CO_2_ (Figure 9C), the performance benefit of STF-Ni was still maintained, though the relative improvement was dramatically reduced, with a narrow increase in maximum current density of 30 mA/cm^2^ in SOFC mode and 40 mA/cm^2^ in SOEC mode. Considering that the STF electrode was active in both reactive environments, these results clearly indicate that the parent perovskite is primarily responsible for establishing the electrocatalytic performance of the electrode. The impedance spectra (Figure 9B and D) show that the ohmic resistance (*R*_ohm_) was always larger on the STF-based cell than on the STF-Ni cell (Table 4), which suggests that STF-Ni had higher electronic conductivity because of the exsolved metal nanoparticles. Since the cells were supported by the electrolyte (150 μm thick), *R*_ohm_ was also larger than the polarization resistance (*R*_pol_) under all the explored conditions and constituted the main contributor to the cells’ performance in both fuel cell and electrolysis mode. Due to the formation of carbonates, and to the more oxidized state of the perovskites’ surface, *R*_ohm_ was higher in CO/CO_2_ than in humidified H_2_. Coming to the electrodic processes, *R*_pol_ was smaller on STF-Ni than on STF: considering that the cells mounted the same oxygen electrode (15 μm thick, 35% porous LSCF-GDC), this difference in *R*_pol_ was associated with the kinetics of the fuel electrode reactions. In this respect, the Bode plots show the presence of two peaks, a main one located at 1–0.5 Hz, and a second at 30–50 Hz: the latter peak was more evident in humidified H_2_, and significantly decreased passing from STF to STF-Ni; the former peak was instead observed both on STF and STF-Ni regardless of the supplied mixture. This observation indicates that the peak at lower frequency was related to the electrocatalytic activity of the parental perovskite, which also contributed to the largest portion of *R*_pol_, as evidenced by the area subtended by the corresponding arc. The fact that the STF and STF-Ni electrodes were both thin and porous (30 μm, also 35% porous) allows us to associate the impedance features with the kinetic processes, rather than to mass diffusion, despite their low frequencies. Consistently with the interpretations reported by Melcher et al.^41^ on Fe exsolving electrodes, the almost overlapping performances of STF-Ni and STF in CO_2_ electrolysis would stem from the fact that this reaction entirely takes place on the parent perovskite’s surface via carbonate intermediates, and does not require any associative desorption step for CO which involves the metal nanoparticle. In the present case, the almost matching performances observed in the fuel cell mode suggest that also the CO conversion mechanism occurs on the perovskite rather than on the exsolved nanoparticles. Under reducing conditions, the role of the exsolved Ni–Fe nanoparticles was likely just to extend the electrocatalytically active surface area (6 m^2^/g in STF-Ni vs 4 m^2^/g in STF, Figure S8), and improve the electronic conductivity. Under more oxidizing conditions (at 750 °C, pO_2_ is 1.77 × 10^–20^ bar in the 50% CO 50% CO_2_ mixture vs 3.62 × 10^–23^ bar in the 97% H_2_ 3% H_2_O mixture), the limited performance difference suggests that the compositional modifications at surface level and the variation of the oxidation state of the reducible B-cations (Sections 3.1 and 3.2) led to a comparable state, with a much less prominent role for the nanoparticles. The major benefit of exsolution in STF-Ni emerged under long-term operation, wherein 100 h stable conduction was achieved in a H_2_/H_2_O mixture, while progressive and irreversible decay was observed for STF.^27^ Hence, the superiority of STF-Ni mainly stems from the stabilization of the performance rather than from its absolute boost. Differently, under CO_2_-rich atmospheres, as during conduction with the CO/CO_2_ mixture, degradation was critical for both materials, and stabilization could not be achieved. As a matter of fact, the in situ characterization and the TEM analysis carried out in this work (Figure 8) clarify that the formation of SrCO_3_ was mostly responsible for the degradation. Thermodynamic calculations show in fact that no coke is expected to form with a 50/50 CO/CO_2_ mixture at 750 °C (the predicted activity of solid carbon *a*_c_ is <1, assuming Δ*G* ^0^_R_ = −170544 + 174.305 · *T* J/mol for the Boudouard reaction^42^). Additionally, the conversion of CO_2_ remained very limited at 1.4 V and −450 mA/cm^2^ (5.5%), which guaranteed that the highest achieved pCO still allowed the fuel electrode to work safely, without coke deposition. As STF was also observed to deactivate more rapidly than STF-Ni in CO/CO_2_,^27^ a detrimental role of segregated Fe particles could be assumed.^41^

**Table 4:** *R*_ohm_ and *R*_pol_ Were Measured on the STF-Based Cell and on the STF-Ni-Based Cell in the Experiments with 3% Humidified H_2_ and with the 50/50 CO/CO_2_ Mixture at 750 °C^a^

	*R*_ohm_ [Ω cm^2^]	*R*_pol_ [Ω cm^2^]	F.E. supply
**STF**	0.58	0.23	3% humidified H_2_
**STF-Ni**	0.49	0.21	
**STF**	0.69	0.42	50/50 CO/CO_2_
**STF-Ni**	0.65	0.37	

a The impedance spectra were collected at the OCV. F.E. indicates fuel electrode.

**Figure 9:**
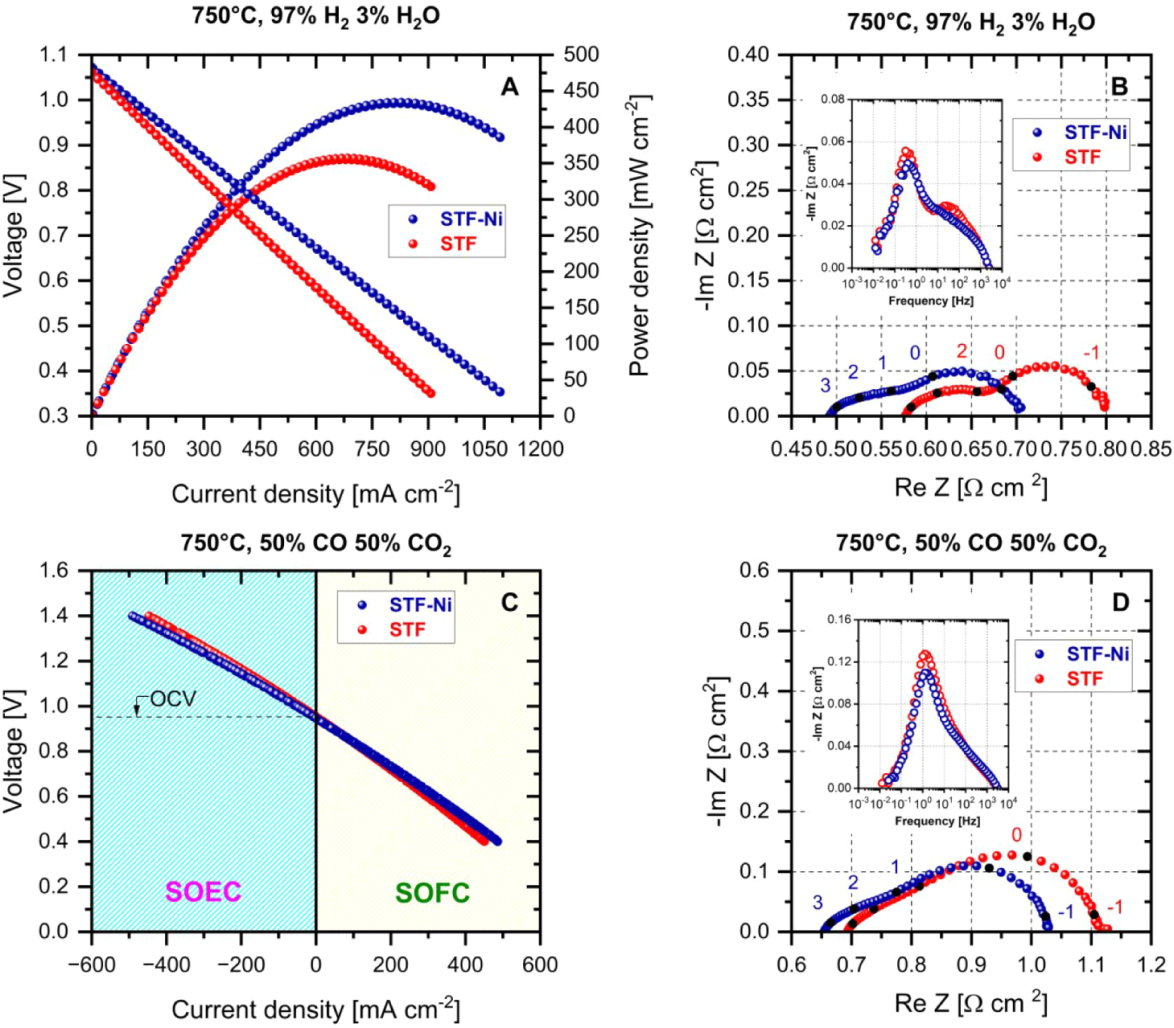
*I*/*V* curves, impedance spectra, and Bode plots of STF and STF-Ni electrode-based cells in 3% humidified H_2_ SOFC mode operation (panels A and B) and 50%CO/50%CO_2_ reversible operation (panels C and D). The EIS measurements are collected at the OCV. The logarithm of the frequency decade is indicated.

## 4 Discussion

The results of the characterization of STF and STF-Ni underscore how the introduction of A-site substoichiometry and a subtle change in B-site composition, namely, the inclusion of Ni, reshapes the redox dynamics and the electrochemical behavior of perovskite fuel electrodes. The XRD patterns collected under 5% H_2_ clearly show that Ni doping promotes rapid and extensive exsolution of metallic nanoparticles. In STF-Ni, alloyed FeNi and FeNi_3_ phases form early during heating (450 °C, Figure 1) and keep evolving during the isothermal hold at 750 °C. The FeNi alloy content increases from 1.48 wt % (Table 1) at the beginning of the dwell to 1.76 wt % by the end, accompanied by a simultaneous decrease in FeNi_3_ content. This evolution indicates a compositional preference toward equimolar Fe–Ni alloy formation under prolonged reduction. This 1:1 Fe-to-Ni ratio is further corroborated by an EDS analysis performed on the exsolved nanoparticles after reduction (Figure S7). Additionally, the refined content of metallic Ni increases to 2.02 wt % (Table 2), suggesting that not all Ni participates in alloying. Overall, the amount of metallic phases in STF-Ni corresponds to approximately 4 wt % in comparison to the amount of Fe present in the bulk of STF (1.42 wt %, Table 1), where segregation of Fe particles occurs, though to a smaller extent. A minor secondary phase of SrO is detected exclusively in STF-Ni (1.48 wt %, Table 2) under 5% H_2_, while it remains undetectable in undoped STF. This implies that Ni incorporation enhances the B-site reducibility with a corresponding modification of the surface-to-bulk energy within the BO_6_ octahedra, promoting partial segregation of A-site cations toward the surface.^40^ The structural changes are hence not only more extensive in STF-Ni, but also more complex since they involve multiple metallic phases and simultaneous SrO reduction. Notably, SrO formation does not depend solely on A-site deficiency (as STF-Ni was purposely prepared as Sr-deficient), but instead appears to be associated with the presence of Ni and its impact on oxygen vacancy formation and lattice strain.^32^ In fact, the onset of SrO in STF-Ni at temperatures where the perovskite structure remains cubic (750 °C, Figure 1) suggests that B-site exsolution perturbs the local environment enough to trigger A-site migration and eventual phase separation.

The irreversible nature of these structural modifications becomes evident during reoxidation. While STF regains its initial phase purity and reincorporates metallic Fe into the lattice without passing from intermediate FeO_*x*_ species, STF-Ni exhibits incomplete redissolution of the exsolved Ni nanoparticles, while Fe redissolution in the oxide matrix passes from Fe_3_O_4_ intermediates. Instead of full reintegration, part of the exsolved Ni reoxidizes to NiO and stabilizes at the surface due to Sr surface enrichment (Figure 5). Instead of reintegration, Ni reoxidizes to NiO and stabilizes at the surface, accompanied by the partial persistence of SrO. This piece of evidence suggests that Ni, once reduced and exsolved, becomes spatially decoupled from the perovskite lattice, a feature that is consistent with the lower solubility of NiO compared to Fe. The partial reversibility of Fe and Ni in STF-Ni thus introduces a degree of structural memory in the material, which may either stabilize active sites under reducing conditions or lead to partial loss of performance enhancement under oxidizing atmospheres due to the loss of reduced Fe. These bulk-level observations are mirrored at the surface by in situ XPS and TEM. Under H_2_, the chemical shift of Sr 3d and O 1s signals in STF-Ni indicates a stronger perturbation to the electronic structure, which is consistent with a higher concentration of oxygen vacancies and with a more reduced surface. The drop in surface oxygen and the increase in surface Sr support a scenario wherein Ni doping destabilizes the perovskite surface by enhancing reducibility and promoting cation segregation. During exposure to CO_2_, both XPS and TGA reveal a marked difference between the two materials. STF-Ni undergoes a larger CO_2_ uptake, which aligns with a higher oxygen vacancy concentration. A recovery of surface oxygen and an increase in the Sr surface component are observed in XPS, indicating that surface-bound SrO readily transforms into SrCO_3_. This is further confirmed by TEM-EDS, which identifies Sr–C–O surface films on STF. Moreover, in STF-Ni, while Fe reincorporates into the bulk during CO_2_ exposure, Ni remains surface-bound, likely as NiO, as observed during oxidation under 10% O_2_ with the XRD experiments. This compositional variation further reinforces the idea that Fe remains coupled to the perovskite lattice, while Ni, once exsolved, mostly remains on the surface. These structural and chemical evolutions directly affect the electrochemical performance of the two electrodes. Under SOFC conditions, STF-Ni delivers higher current densities and power output than STF. However, under more oxidizing regimes, such as in reversible CO/CO_2_ operation, the performance gap narrows. This narrowed margin suggests that the oxidation of surface nanoparticles and the formation of carbonate reduce the effectiveness of the exsolved phases, a conclusion supported by the persistence of NiO and SrCO_3_. Interestingly, the redox-sensitive surface of STF-Ni also enables some degree of regeneration, especially under alternating redox cycling, which may periodically reactivate surface species and mitigate degradation. Altogether, these findings emphasize a complex balance: Ni doping enables faster reduction, enhanced exsolution, and higher initial activity but introduces irreversible surface modifications and redox behavior asymmetry. Importantly, the partial regeneration of its surface properties under reversible SOC’s conduction highlights a self-adaptive behavior that could be harnessed through optimized cycling (regeneration) strategies.

## 5 Conclusions

This study explores the redox behavior, surface chemistry, and electrochemical performance of sub-STF-Ni and its undoped counterpart STF, under conditions relevant to applications as SOCs’ fuel electrodes. By integrating in situ XRD, in situ NAP-XPS, TGA, TEM, and electrochemical testing, we tracked the structural evolution and functional properties of these materials. The use of synchrotron-based in situ techniques provided a time-resolved view of the dynamic transformations occurring under redox cycling and reactive gas exposure, offering insights into phase stability, surface evolution, and exsolution behavior that are not accessible via conventional ex situ analysis. The following key findings highlight the potential of STF-Ni as an advanced fuel electrode material:

- In situ XRD and NAP-XPS experiments show that Sr-deficient STF-Ni begins exsolving bimetallic Fe–Ni nanoparticles already at 450 °C. Comparatively, STF segregates larger metal Fe particles only at a higher temperature (750 °C).
- The Rietveld analyses of the XRD patterns of STF-Ni confirm the complete reintegration of Fe from the Fe–Ni nanoparticles into the perovskite lattice upon reoxidation in 10% O_2_, whereas Ni persists primarily as NiO, indicating asymmetric reincorporation behavior of the exsolved metals.
- TEM studies confirm the compositional evolution of exsolved nanoparticles, revealing that they primarily consist of Fe and Ni in the reduced state, while exposure to CO_2_ results in Ni-rich nanoparticles with increased size. A few Fe nanoparticles and larger aggregates are also found in the STF after reduction.
- Exposure to CO_2_ leads to the formation of carbonate and surface reoxidation in both perovskites.
- When applied as SOC’s fuel electrodes, the performance differences between STF-Ni and STF narrow under oxidizing feeds due to nanoparticle oxidation and surface passivation.

## Supplementary Material



## Notes

XRD patterns of the as-prepared STF powders; cell parameters of STF and STF-Ni samples calculated under reducing (5% H_2_ 95% N_2_) and oxidizing conditions (10% O_2_ 90% N_2_); in situ XRD patterns of STF and STF-Ni samples collected during the exposure to reducing conditions (240 min, 750 °C) and during the exposure to oxidizing conditions (64 min, 750 °C); TEM images of STF powders, as-prepared, after reduction, and after exposure to CO_2_; TEM images of STF-Ni nanoparticles after exposure to CO_2_; EDS elemental composition maps of Fe–Ni exsolved nanoparticles, STF-Ni bulk areas and STF bulk areas; BET isotherms of STF-Ni and STF powders after reduction (PDF)

## References

[1] Neagu D., Irvine J. T. S., Wang J., Yildiz B., Opitz A. K., Fleig J., Wang Y., Liu J., Shen L., Ciucci F. (2023). Roadmap on Exsolution for Energy Applications. J. Phys. Energy.

[2] Kim Y. H., Jeong H., Won B.-R., Jeon H., Park C., Park D., Kim Y., Lee S., Myung J. (2024). Nanoparticle
Exsolution on Perovskite Oxides: Insights into Mechanism, Characteristics
and Novel Strategies. Nano-Micro Lett..

[3] Kwon O., Joo S., Choi S., Sengodan S., Kim G. (2020). Review on Exsolution
and Its Driving Forces in Perovskites. J. Phys.
Energy.

[4] Nishihata Y., Mizuki J., Akao T., Tanaka H., Uenishi M., Kimura M., Okamoto T., Hamada N. (2002). Self-Regeneration of
a Pd-Perovskite Catalyst for Automotive Emissions Control. Nature.

[5] Steiger P., Kröcher O., Ferri D. (2020). Increased Nickel Exsolution from
LaFe0.8Ni0.2O3 Perovskite-Derived CO2Methanation Catalysts through
Strontium Doping. Appl. Catal. A Gen..

[6] Cho E., Lee Y.-H., Kim H., Jang E. J., Kwak J. H., Lee K., Ko C. H., Yoon W. L. (2020). Ni Catalysts for Dry Methane Reforming
Prepared by A-Site Exsolution on Mesoporous Defect Spinel Magnesium
Aluminate. Appl. Catal. A Gen..

[7] Wei T., Jia L., Zheng H., Chi B., Pu J., Li J. (2018). LaMnO3-Based
Perovskite with in-Situ Exsolved Ni Nanoparticles: A Highly Active,
Performance Stable and Coking Resistant Catalyst for CO2 Dry Reforming
of CH4. Appl. Catal. A Gen..

[8] Kim Y. H., Kang Y., Jo S., Jeong H., Neagu D., Myung J. (2022). Shape-Shifting Nanoparticles
on a Perovskite Oxide for Highly Stable
and Active Heterogeneous Catalysis. Chem. Eng.
J..

[9] Schrenk F., Lindenthal L., Drexler H., Urban G., Rameshan R., Summerer H., Berger T., Ruh T., Opitz A. K., Rameshan C. (2022). Impact of
Nanoparticle Exsolution on Dry Reforming
of Methane: Improving Catalytic Activity by Reductive Pre-Treatment
of Perovskite-Type Catalysts. Appl. Catal. B
Environ..

[10] Neagu D., Oh T.-S., Miller D. N., Ménard H., Bukhari S. M., Gamble S. R., Gorte R. J., Vohs J. M., Irvine J. T. S. (2015). Nano-Socketed Nickel Particles with
Enhanced Coking
Resistance Grown in Situ by Redox Exsolution. Nat. Commun..

[11] Mukhopadhyay M., Mukhopadhyay J., Sharma A. D., Basu R. N. (2011). In-Situ Patterned
Intra-Anode Triple Phase Boundary in SOFC Electroless Anode: An Enhancement
of Electrochemical Performance. Int. J. Hydrogen
Energy.

[12] Schwiers A., Röhrer D., Lenser C., Steinrücken B., Sebold D., Spliethoff H., Guillon O., Menzler N. H. (2024). Phase Stability,
Redox-Behavior and Carbon-Tolerance of Sr1–x­(Ti0.3Fe0.7–yNiy)­O3−δ
with Exsolved Nanoparticles. J. Mater. Chem.
A.

[13] Neagu D., Tsekouras G., Miller D. N., Ménard H., Irvine J. T. S. (2013). In Situ Growth of Nanoparticles through Control of
Non-Stoichiometry. Nat. Chem..

[14] Zhu T., Troiani H., Mogni L. V., Santaya M., Han M., Barnett S. A. (2019). Exsolution and Electrochemistry
in Perovskite Solid
Oxide Fuel Cell Anodes: Role of Stoichiometry in Sr­(Ti,Fe,Ni)­O3. J. Power Sources.

[15] Han F.-Z., Wan Y.-F., Li C.-X., Zhang S.-L. (2024). Sr­(Ti0·3Fe0.7)­O3−δ-Based
Perovskite with in-Situ Exsolved Fe–Ru Nanoparticles: A Highly
Stable Fuel Electrode Material for Solid Oxide Electrochemical Cells
with Efficient Electrocatalytic CO2 Reduction Ability and Preferential
Selectivity. J. Power Sources.

[16] Donazzi A., Schmauss T. A., Barnett S. A. (2022). Catalytic
and Electrocatalytic Performance
of Sr­(Ti0.3Fe0.7Ru0.07)­O3-δ for Applications in Solid Oxide
Fuel Cells Supplied with Ethanol Steam Reforming Mixtures. J. Power Sources.

[17] Santaya M., Jiménez C. E., Troiani H. E., Carbonio E. A., Arce M. D., Toscani L. M., Garcia-Diez R., Wilks R. G., Knop-Gericke A., Bär M., Mogni L. V. (2022). Tracking the Nanoparticle Exsolution/Reoxidation
Processes of Ni-Doped SrTi0.3Fe0.7O3−δ Electrodes for
Intermediate Temperature Symmetric Solid Oxide Fuel Cells. J. Mater. Chem. A.

[18] Zhang S.-L., Wang H., Yang T., Lu M. Y., Li C.-X., Li C.-J., Barnett S. A. (2020). Advanced
Oxygen-Electrode-Supported
Solid Oxide Electrochemical Cells with Sr­(Ti,Fe)­O3−δ-Based
Fuel Electrodes for Electricity Generation and Hydrogen Production. J. Mater. Chem. A.

[19] Glaser R., Zhu T., Troiani H., Caneiro A., Mogni L., Barnett S. (2018). The Enhanced
Electrochemical Response of Sr­(Ti0.3Fe0.7Ru0.07)­O3−δ
Anodes Due to Exsolved Ru–Fe Nanoparticles. J. Mater. Chem. A.

[20] Zhu T., Troiani H. E., Mogni L. V., Han M., Barnett S. A. (2018). Ni-Substituted
Sr­(Ti,Fe)­O3 SOFC Anodes: Achieving High Performance via Metal Alloy
Nanoparticle Exsolution. Joule.

[21] Reinke J. M., Barnett S. A. (2025). Mapping Phase Instability
to Electrochemical Degradation
in SrTi1–xFexO3−δ under Solid Oxide Cell Fuel-Electrode
Conditions. J. Mater. Chem. A.

[22] Carrillo A.
J., López-García A., Delgado-Galicia B., Serra J. M. (2024). New Trends in Nanoparticle Exsolution. Chem. Commun..

[23] Weber M. L., Sohn Y. J., Dittmann R., Waser R., Menzler N. H., Guillon O., Lenser C., Nem̆ák S., Gunkel F. (2023). Reversibility Limitations of Metal
Exsolution Reactions
in Niobium and Nickel Co-Doped Strontium Titanate. J. Mater. Chem. A.

[24] Katz M. B., Zhang S., Duan Y., Wang H., Fang M., Zhang K., Li B., Graham G. W., Pan X. (2012). Reversible
Precipitation/Dissolution of Precious-Metal Clusters in Perovskite-Based
Catalyst Materials: Bulk versus Surface Re-Dispersion. J. Catal..

[25] Opitz A. K., Nenning A., Vonk V., Volkov S., Bertram F., Summerer H., Schwarz S., Steiger-Thirsfeld A., Bernardi J., Stierle A., Fleig J. (2020). Understanding Electrochemical
Switchability of Perovskite-Type Exsolution Catalysts. Nat. Commun..

[26] Myung J., Neagu D., Miller D. N., Irvine J. T. S. (2016). Switching
on
Electrocatalytic Activity in Solid Oxide Cells. Nature.

[27] Lacharme M. C. D., Donazzi A. (2025). Characterization and Testing of Strontium Titanium
Ferrite-Based Solid Oxide Cells for Reversible and Co-Electrolysis
Operation. J. Power Sources.

[28] Pagliari M., Marasi M., Donazzi A. (2024). Electrocatalytic
and Operando Characterization
of State-of-the-Art SOFC Cathodes for Applications at High CO2 Concentration
in Novel Clean Power Production Cycles. Electrochim.
Acta.

[29] Orsini F., Ferrero D., Cannone S. F., Santarelli M., Felli A., Boaro M., de Leitenburg C., Trovarelli A., Llorca J., Dimitrakopoulos G., Ghoniem A. F. (2023). Exsolution-Enhanced Reverse Water-Gas Shift Chemical
Looping Activity of Sr2FeMo0.6Ni0.4O6-δ Double Perovskite. Chem. Eng. J..

[30] Mroziński A., Molin S., Błaszczak P., Miruszewski T., Górnicka K., Karczewski J., Jasiński P. (2023). Impact of
Strontium Non-Stoichiometry of SrxTi0.3Fe0.7O3-δ on Structural,
Electrical, and Electrochemical Properties for Potential Oxygen Electrode
of Intermediate Temperature Solid Oxide Cells. Int. J. Hydrogen Energy.

[31] Görke R. H., Marek E. J., Donat F., Scott S. A. (2020). Reduction and Oxidation
Behavior of Strontium Perovskites for Chemical Looping Air Separation. Int. J. Greenh. Gas Control..

[32] Koo B., Kim K., Kim J. K., Kwon H., Han J. W., Jung W. (2018). Sr Segregation
in Perovskite Oxides: Why It Happens and How It Exists. Joule.

[33] Crumlin E. J., Mutoro E., Liu Z., Grass M. E., Biegalski M. D., Lee Y.-L., Morgan D., Christen H. M., Bluhm H., Shao-Horn Y. (2012). Surface Strontium Enrichment on Highly
Active Perovskites
for Oxygen Electrocatalysis in Solid Oxide Fuel Cells. Energy Environ. Sci..

[34] Opitz A. K., Rameshan C., Kubicek M., Rupp G. M., Nenning A., Götsch T., Blume R., Hävecker M., Knop-Gericke A., Rupprechter G., Klötzer B., Fleig J. (2018). The Chemical Evolution
of the La0.6Sr0.4CoO3−δ Surface
Under SOFC Operating Conditions and Its Implications for Electrochemical
Oxygen Exchange Activity. Top. Catal..

[35] Nenning A., Opitz A. K., Rameshan C., Rameshan R., Blume R., Hävecker M., Knop-Gericke A., Rupprechter G., Klötzer B., Fleig J. (2016). Ambient Pressure XPS Study of Mixed
Conducting Perovskite-Type SOFC Cathode and Anode Materials under
Well-Defined Electrochemical Polarization. J.
Phys. Chem. C.

[36] Rothschild A., Menesklou W., Tuller H. L., Ivers-Tiffée E. (2006). Electronic
Structure, Defect Chemistry, and Transport Properties of SrTi1-XFexO3-y
Solid Solutions. Chem. Mater..

[37] Deng X., Verdaguer A., Herranz T., Weis C., Bluhm H., Salmeron M. (2008). Surface Chemistry
of Cu in the Presence of CO2 and
H2O. Langmuir.

[38] Jiang Y., Liu J., Cheng B., Dang X., Su H., Hua Y., Gao Z. (2024). In Situ Exsolved
NiFe Nanoparticles in Ni-Doped Sr0.9Ti0.3Fe0.63Ni0.07O3-δ
Anode with a Three-Dimensionally Ordered Macroporous Structure for
Solid Oxide Fuel Cells Fueled by Alkanes. Chem.
Eng. J..

[39] Jung W., Tuller H. L. (2012). Investigation of
Surface Sr Segregation in Model Thin
Film Solid Oxide Fuel Cell Perovskite Electrodes. Energy Environ. Sci..

[40] Santaya M., Jiménez C. E., Arce M. D., Carbonio E. A., Toscani L. M., Garcia-Diez R., Knop-Gericke A., Mogni L. V., Bär M., Troiani H. E. (2023). Exsolution versus Particle Segregation on (Ni,Co)-Doped
and Undoped SrTi0.3Fe0.7O3-δ Perovskites: Differences and Influence
of the Reduction Path on the Final System Nanostructure. Int. J. Hydrogen Energy.

[41] Melcher C., Nenning A., Schrenk F., Rath K., Rameshan C., Opitz A. K. (2025). The Dark Side of
Metal Exsolution: A Combined in Situ
Surface Spectroscopic and Electrochemical Study on Perovskite-Type
Cathodes for High-Temperature CO2 Electrolysis. EES Catal..

[42] Huang, K. ; Goodenough, J. B. 1 - Introduction to solid oxide fuel cells (SOFCs). In Solid Oxide Fuel Cell Technology: Principles, Performance and Operations; Woodhead Publishing Series in Energy, 2009; pp. 1-9. DOI: 10.1533/9781845696511.1.

