# Supplementary material for: In Situ Characterization of Strontium Titanium Ferrite Perovskites for Application as Electrodes of Solid Oxide Cells
Source: ACS Appl Energy Mater. 2026 Jan 23;9(3):1508–22. doi: 10.1021/acsaem.5c03226 (PMC12892881; doi:10.1021/acsaem.5c03226)
Supplement: Supplementary file 1 [file ae5c03226_si_001.pdf]

**Supporting Information**

# In situ characterization of strontium titanium ferrite perovskites for application as electrodes of solid oxide cells

Maria Carmenza Diaz Lacharme[a], Martina Marasi[a], Virginia Pérez Dieste[b], Belen Ballesteros[c], Alessandro Donazzi[a]*

[a] Department of Energy, Politecnico di Milano, via Lambruschini 4, 20156, Milan, Italy

[b] ALBA Synchrotron Light Source, Carrer de la Llum 2-26, 08290 Cerdanyola del Vallès, Barcelona, Spain

[c] Catalan Institute of Nanoscience and Nanotechnology (ICN2), CSIC and The Barcelona Institute of Science and Technology, Campus UAB, Bellaterra, Barcelona, 08193, Spain

*Corresponding author: alessandro.donazzi@polimi.it

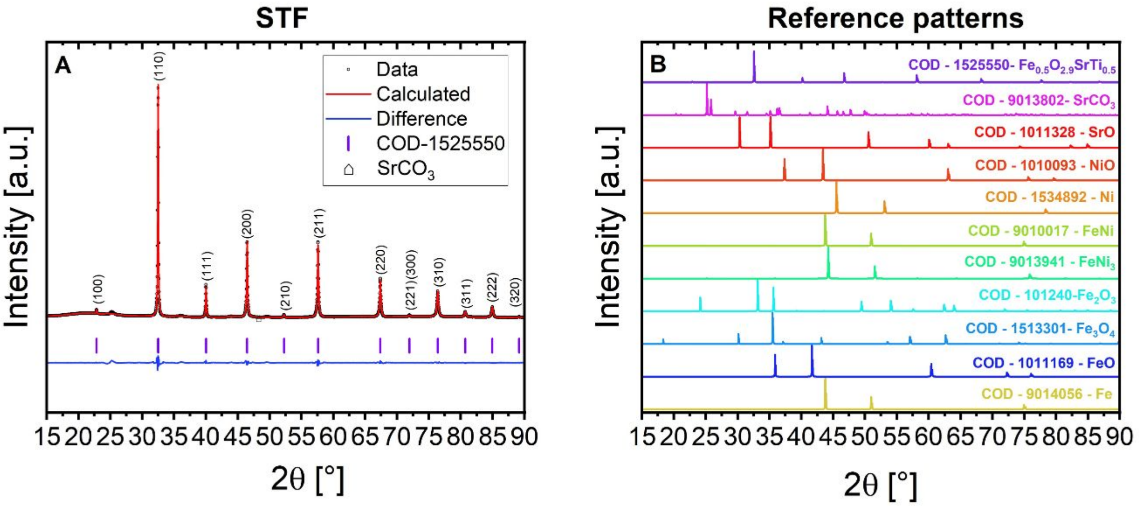


**Figure S1** – (A) XRD patterns of the as-prepared STF powders: observed XRD fingerprint (black symbols), calculated XRD pattern from Rietveld refinement (red line), the difference between observed and calculated intensities (blue line) and tick marks represent reflections of refined phase. (B) Reference XRD Patterns for general phase identification. VESTA software was used for pattern generation according to CIF files of each COD-listed phase.

**Table S1** – Cell parameters of the STF samples during the in situ experiments for reducing (5% $H_2$) and oxidizing conditions (10% $O_2$).

| T [°C] | Mixture | a = b = c [Å] | α = β = γ [°] | Lattice symmetry | V [Å$^3$] | ρ [g cm$^{-3}$] |
|---|---|---|---|---|---|---|
| 25 | 5% $H_2$ in $N_2$ | 3.889 | 90 | m3m | 58.807 | 5.239 |
| 550 | | 3.948 | 90 | m3m | 61.533 | 5.007 |
| 750 (0 min) | | 3.954 | 90 | m3m | 61.794 | 4.986 |
| 750 (240 min) | | 3.959 | 90 | m3m | 62.052 | 4.965 |
| 25 | 10% $O_2$ in $N_2$ | 3.917 | 90 | m3m | 60.087 | 5.127 |
| 550 | | 3.922 | 90 | m3m | 60.311 | 5.108 |
| 750 (0 min) | | 3.941 | 90 | m3m | 61.198 | 5.034 |
| 750 (64 min) | | 3.941 | 90 | m3m | 61.196 | 5.034 |

**Table S2** – Cell parameters of the STF-Ni samples during the in situ experiments for reducing (5% $H_2$) and oxidizing conditions (10% $O_2$).

| T [°C] | Mixture | a = b = c [Å] | α = β = γ [°] | Lattice symmetry | V [Å$^3$] | ρ [g cm$^{-3}$] |
|---|---|---|---|---|---|---|
| 25 | 5% $H_2$ in $N_2$ | 3.918 | 90 | m3m | 60.166 | 5.126 |
| 750 (0 min) | | 3.945 | 90 | m3m | 61.399 | 4.908 |
| 750 (240 min) | | 3.945 | 90 | m3m | 61.399 | 4.908 |
| 25 | 10% $O_2$ in $N_2$ | 3.889 | 90 | m3m | 58.807 | 5.194 |
| 750 (0 min) | | 3.946 | 90 | m3m | 61.461 | 4.757 |
| 750 (64 min) | | 3.961 | 90 | m3m | 62.131 | 4.706 |

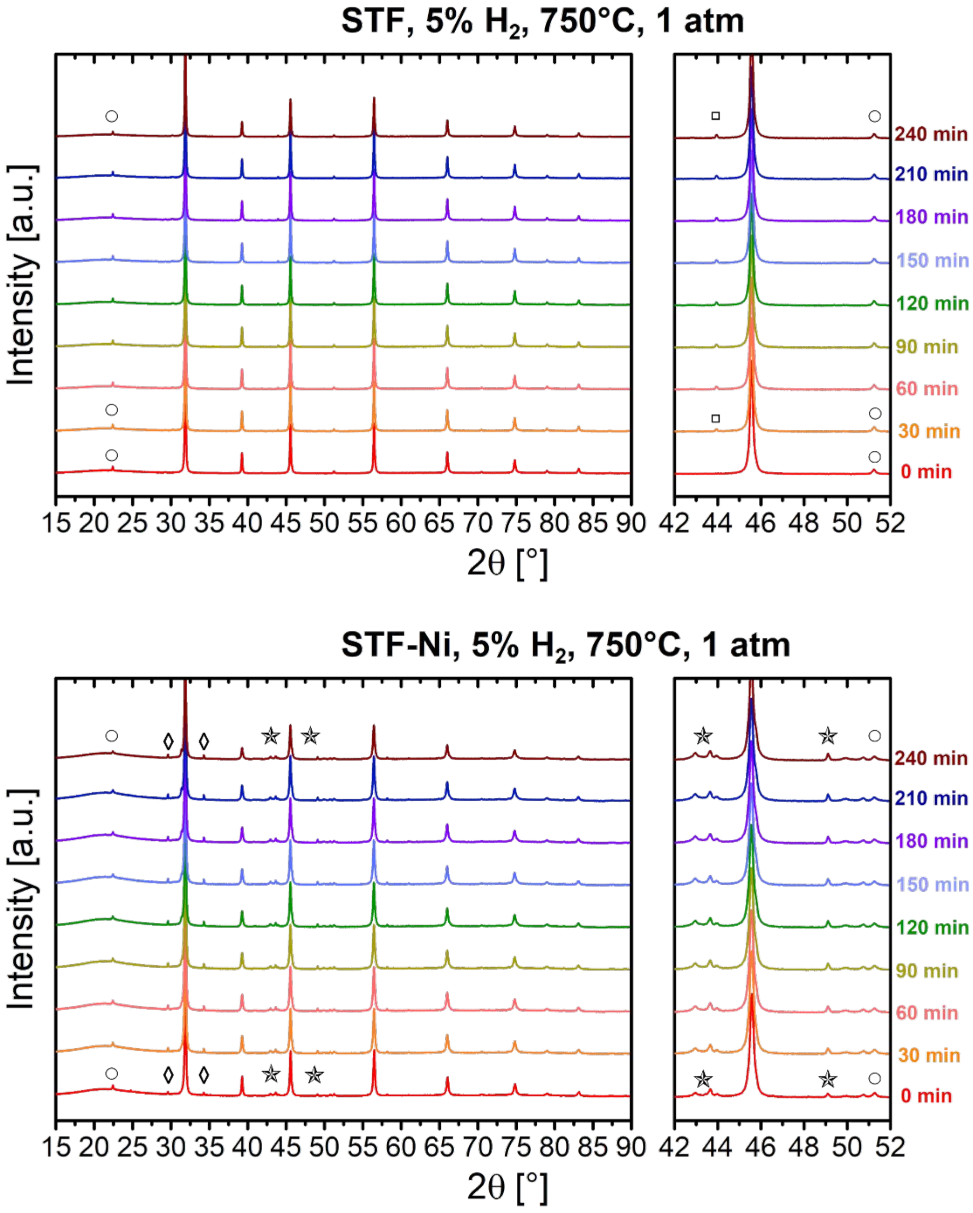


**Figure S2** – In situ XRD patterns of STF (top) and STF-Ni (bottom) collected during the 240 min exposure of the samples to the 5% $H_2$ in $N_2$ mixture at 750°C. Symbols: (⌂) $SrCO_3$, (▪) NiO, (✭) Ni-Fe alloy, (○) $SrTiFeO_3$ phase, (◊) SrO, (▫) Fe.

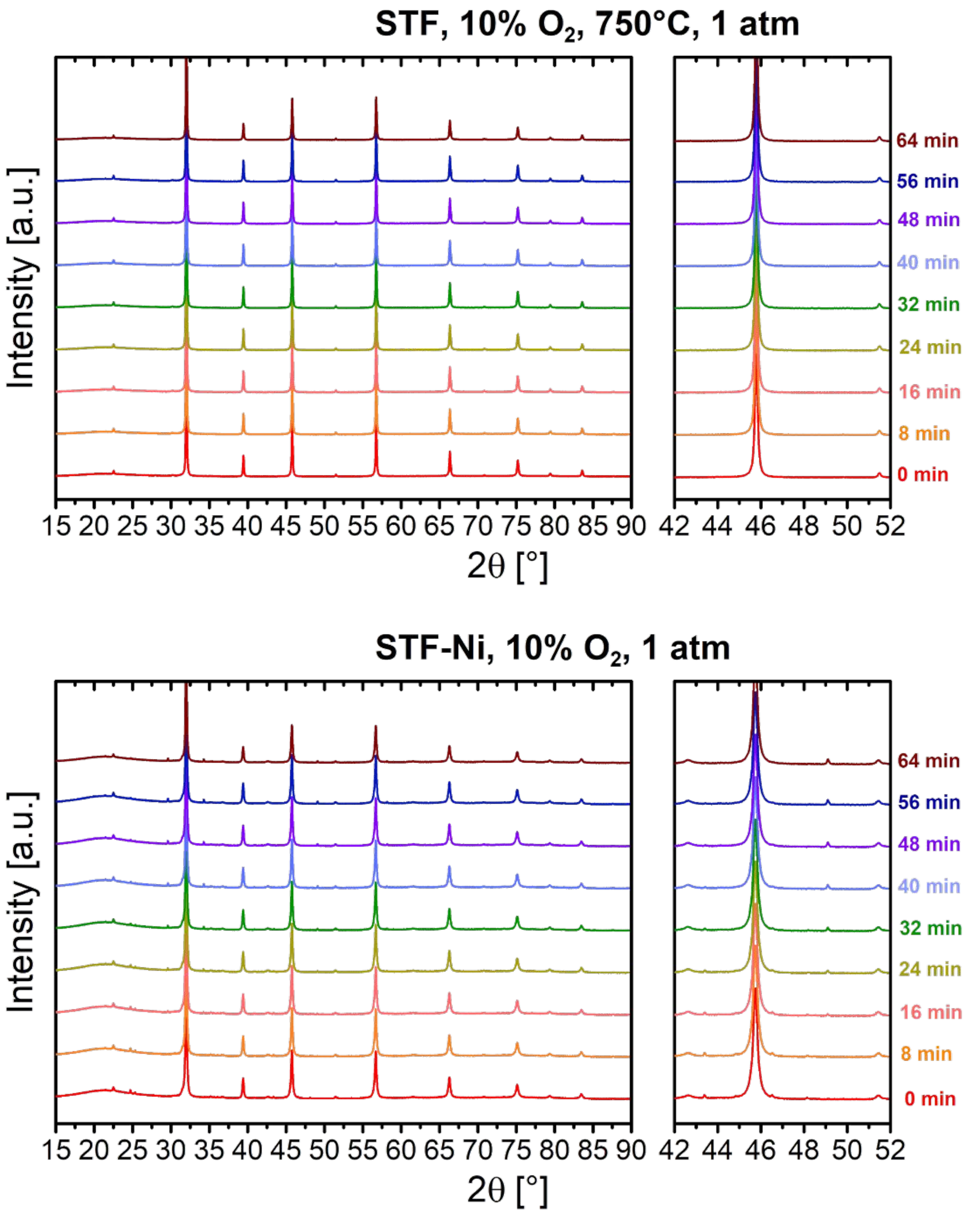


**Figure S3** – In situ XRD patterns of STF (top) and STF-Ni (bottom) collected during the 64 min exposure of the samples to the 10% $O_2$ in $N_2$ mixture at 750°C.

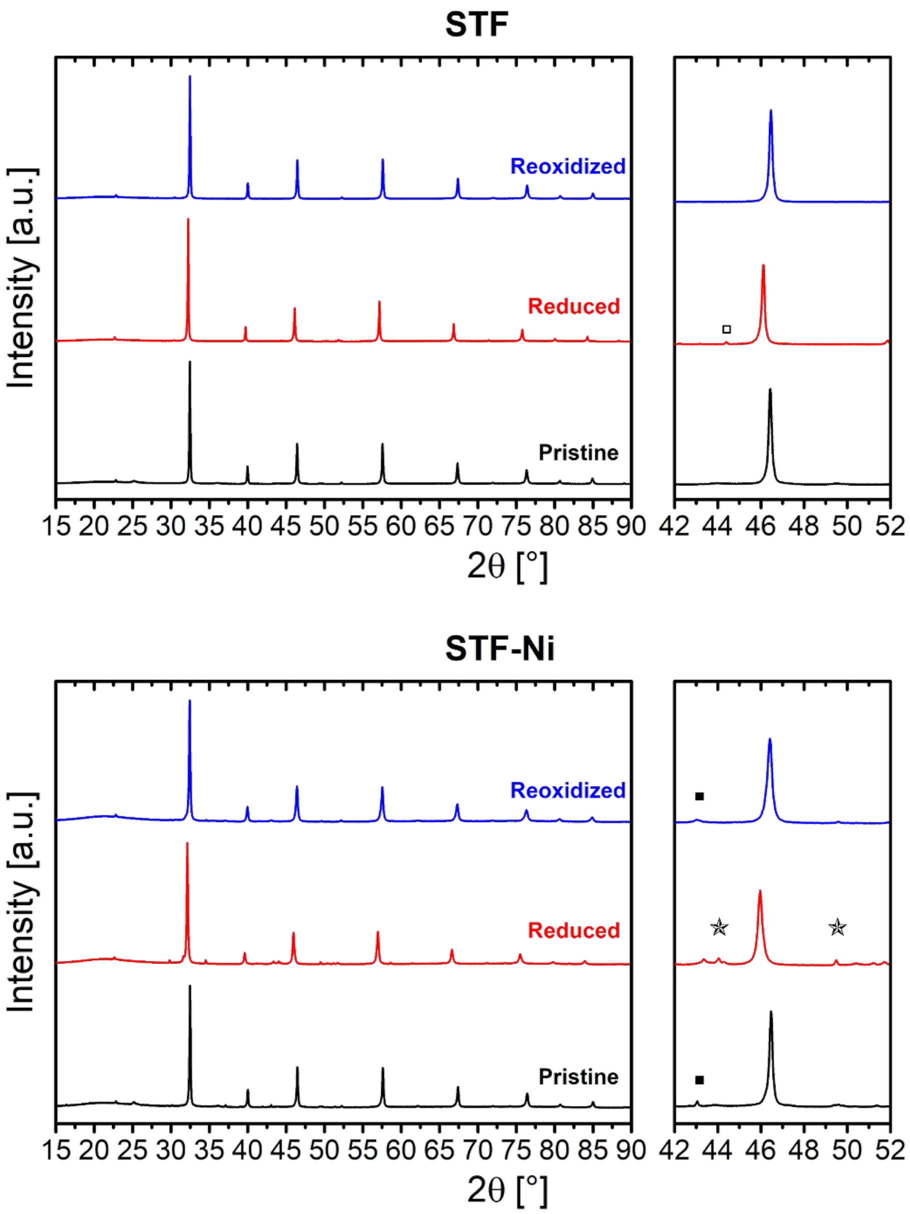


**Figure S4** – Comparison of the XRD patterns of STF (top) and STF-Ni (bottom) at room temperature under pristine, reduced in 5% $H_2$ in $N_2$, and reoxidized in 10% $O_2$ in $N_2$ conditions. Symbols: (⌂) $SrCO_3$, (▪) NiO, (✯) Ni-Fe alloy, (○) $SrTiFeO_3$ phase, (◊) SrO, (▫) Fe.

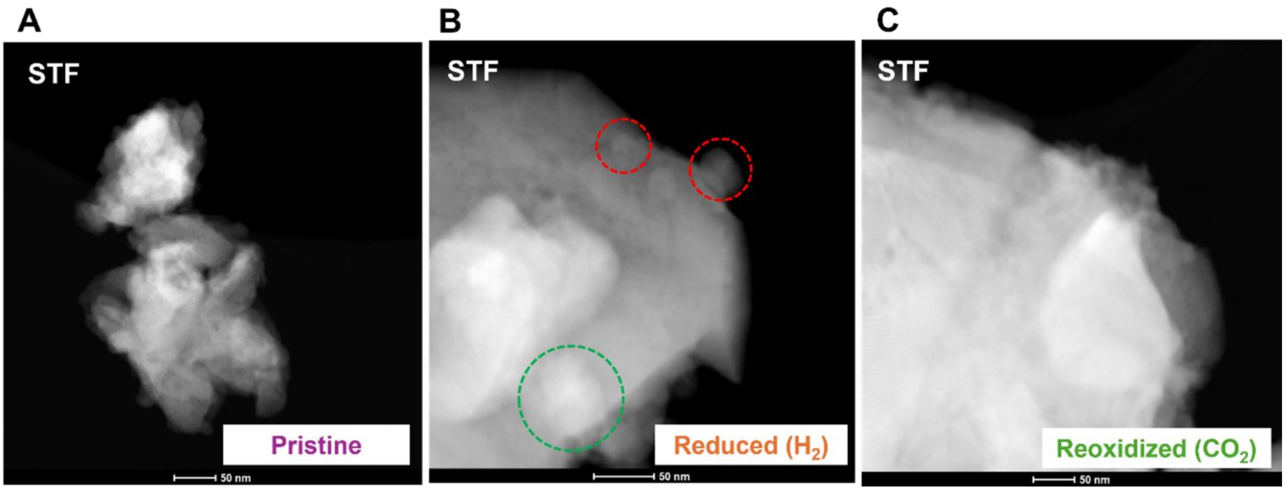


**Figure S5** – TEM images of STF powders appearance under: pristine (as-prepared), reduced in 5% $H_2$, and reoxidized in $CO_2$ conditions. In panel B, nanoparticles and larger aggregates of metal Fe are circled in red and green, respectively.

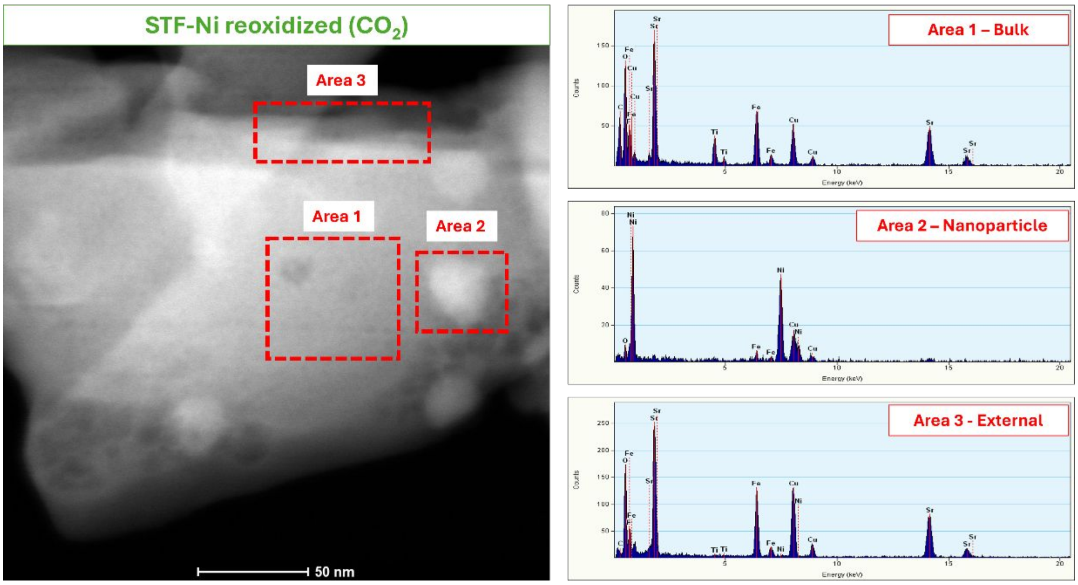


**Figure S6** – TEM image and EDS of STF-Ni nanoparticles after reoxidation in $CO_2$ illustrating changes in elemental distribution. Area 1 represents the perovskite bulk, Area 2 the nanoparticles, and Area 3 an external region exhibiting Sr enrichment.

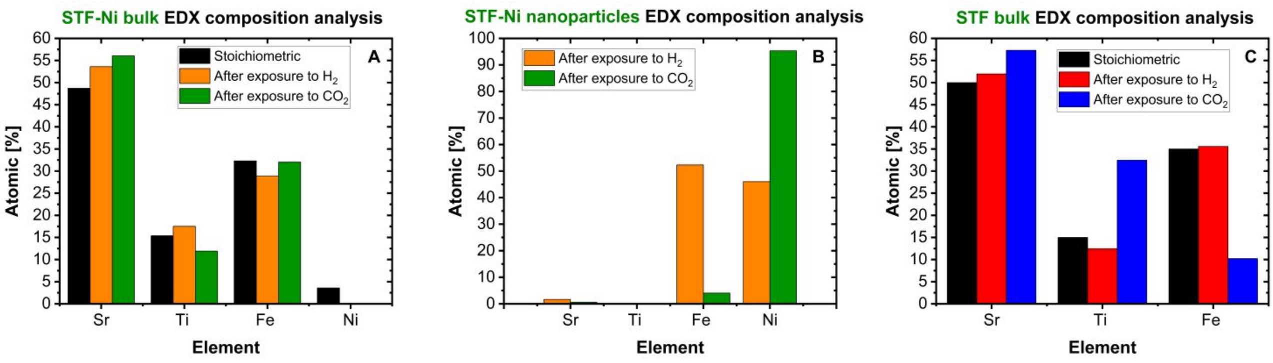


**Figure S7** – Elemental composition as derived from the EDS mapped areas in the case of: STF-Ni bulk (A), Fe-Ni exsolved nanoparticles (B), and STF bulk. The black columns in panels A and C of the scan of the bulk areas represent the elemental fractions expected from the stoichiometric composition of each perovskite.

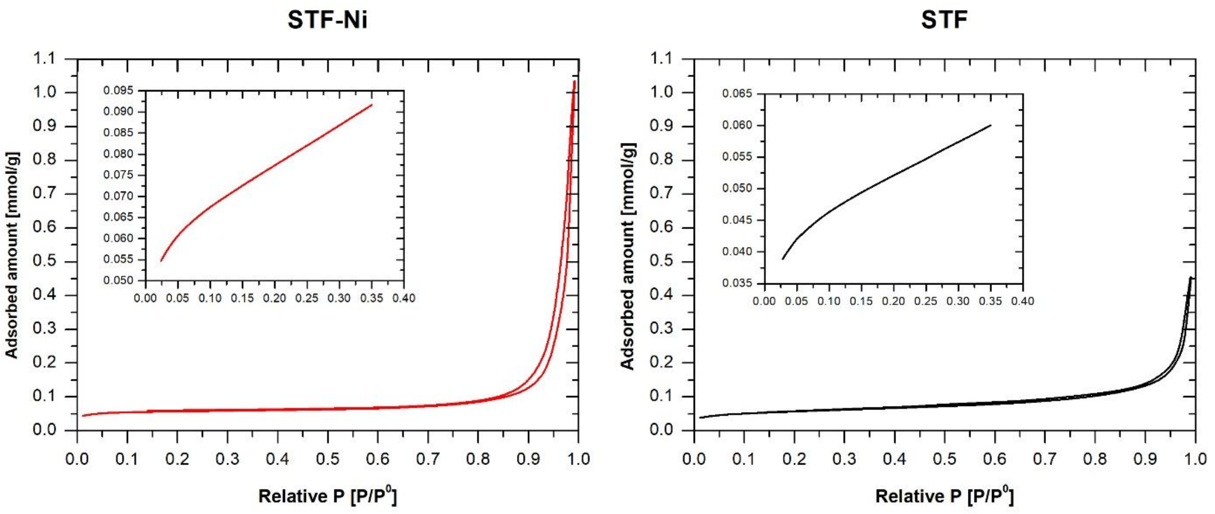


**Figure S8** – Nitrogen adsorption-desorption isotherms of STF-Ni and STF powders after reduction in 5% $H_2$ in Ar at 750°C. In the inserts, a zoom on the B point for the evaluation of the BET surface area of each sample is provided.
